# Quantitative changes in the corneal endothelium and central corneal thickness during anterior chamber inflammation: A systematic review and meta-analysis

**DOI:** 10.1371/journal.pone.0296784

**Published:** 2024-01-05

**Authors:** Germán Mejía-Salgado, Paula Tatiana Muñoz-Vargas, Carlos Cifuentes-González, Gabriela Flórez-Esparza, Rebeca Paquentín-Jiménez, Miguel Ángel Castro-Monreal, Naomi Medina-Galindo, Gilma Norella Hernández-Herrera, Luz Elena Concha-del-Río, Alejandra de-la-Torre

**Affiliations:** 1 Institute of Translational Medicine (IMT), Neuroscience (NEUROS) Research Group, Neurovitae Research Center, Escuela de Medicina y Ciencias de la Salud, Universidad del Rosario, Bogotá, Colombia; 2 Institute of Translational Medicine (IMT), Ophthalmology Interest Group, Neuroscience (NEUROS) Research Group, Neurovitae Research Center, Escuela de Medicina y Ciencias de la Salud, Universidad del Rosario, Bogotá, Colombia; 3 Postgraduate Master’s Program in Epidemiology Universidad CES, Medellín, Colombia; 4 Post-gradual Master’s Program in Epidemiology Universidad del Rosario, Bogotá Colombia; 5 Inflammatory Eye Disease Clinic, Asociación Para Evitar la Ceguera en México, Hospital “Dr. Luis Sánchez Bulnes,” México City, México; University of Warmia, POLAND

## Abstract

**Purpose:**

To establish the effects of anterior chamber inflammation (ACI) on the corneal endothelium parameters and central corneal thickness (CCT).

**Methods:**

We conducted a comprehensive literature review using medical databases (PubMed, EMBASE, VHL, and medRxiv) on March 8, 2023, for studies that included patients with ACI who had undergone specular microscopy or pachymetry. Case series with >10 patients, cross-sectional, case-control, and cohort studies were included. The risk of bias was assessed using CLARITY tools and validated scales such as those by Hassan Murad et al. and Hoy et al. A narrative synthesis and a quantitative standardized mean difference meta-analysis, I^2^ heterogeneity assessment, and publication bias tests were conducted. The study was registered in PROSPERO (CRD42023420148) and approved by the Universidad del Rosario ethical committee (DVO005 2277- CV1712).

**Results:**

Thirty-four studies, encompassing 1,388 eyes with ACI, were included. Compared with healthy controls, overall, ACI eyes show significant mean differences in endothelial parameters (endothelial cell density (ECD), coefficient of variation (CV), and hexagonality (HEX)) (P < 0.05). In the subgroup analysis compared with healthy controls, both active and chronic-recurrent ACI demonstrated a reduced ECD. An increased CV was observed in active, inactive, and chronic-recurrent ACI. Lower HEX was evident in inactive, acute, and chronic-recurrent ACI, while both active and acute ACI exhibited high CCT.

**Conclusion:**

ACI leads to significant alterations in endothelial parameters and CCT. The primary contributors to these changes are increased IOP, uveitis duration, and intraocular surgeries. Further studies are needed to explore the impact of ACI etiology on the endothelium, potential biases in IOP measurements during acute ACI episodes, and the potential necessity for monitoring the endothelial parameters and CCT in patients with chronic ACI.

## Introduction

The corneal endothelium is the posterior monolayer of the cornea, which appears as a honeycomb-like mosaic when viewed from the rear side. Its primary function is maintaining corneal clarity by ensuring it remains relatively deturgesced [1]. Human endothelial cells show no mitotic activity in vivo; however, humans are born with a significant reserve cell density of approximately 3,500 cells/mm^2^, decreasing gradually at approximately 0.6% per year. As endothelial cells get damaged, they lose their mosaic shape, which changes their size (polymegatism) and their characteristic hexagonal shape (pleomorphism) [2].

Anterior chamber inflammation (ACI) can induce corneal complications such as band keratopathy, anterior synechiae, and keratic precipitates [3,4]. Regarding endothelial injury, some studies have highlighted a loss in endothelial cell density (ECD) and hexagonality (HEX), along with higher coefficients of variation (CV) in those with ACI [5–7]. Furthermore, central corneal thickness (CCT) is often increased because of the impaired semipermeable barrier function during acute episodes of ACI [8,9]. Elevated intraocular pressure (IOP) and surgeries addressing uveitis complications, such as cataracts and glaucoma, are considered primary contributors to this endothelial damage [10–13].

Although endothelial damage is not one of the most common complications of uveitis, approximately 1.10% of individuals with ocular inflammation require keratoplasty within the first ten years, with patients with ACI being at higher risk (HR 2.97) [14]. Despite these findings, there is a dearth of studies on quantitative changes in endothelial metrics and CCT. This systematic review aims to quantify changes in endothelial parameters and CCT in patients with ACI, improving the understanding of endothelial damage in intraocular inflammation and accentuating the relevance of imaging techniques such as specular or confocal microscopy in uveitis.

## Materials and methods

### Type and design of the study

Using the Preferred Reporting Items for Systematic Reviews and Meta-Analyses (PRISMA) guidelines, we conducted a systematic literature review and meta-analysis **(S1 File)** [15]. The study was registered in PROSPERO (CRD42023420148) and approved by the Universidad del Rosario ethics committee (DVO005 2277-CV1712).

### Study selection criteria

We included primary observational studies such as case series with > 10 patients, case-control studies, cohort studies, cross-sectional studies, and clinical trials. We excluded nonfull-text texts, studies in species other than humans, case reports, economic analyses, systematic reviews, and meta-analyses. The inclusion criteria were patients of all ethnicities, ages, and genders presenting with ACI (including anterior uveitis, anterior-intermediate uveitis, or panuveitis) of any origin and clinical features with specular microscopy description or pachymetry information. Patients diagnosed with chronic glaucoma and/or cataract surgery before ACI were excluded.

### Sources of information

We conducted the literature search independently in four databases, PubMed, EMBASE, Virtual Health Library (VHL), and medRxiv, covering all available records until March 8, 2023. We used the keywords “uveitis” and “corneal endothelium” among multiple search combinations to achieve the highest sensitivity and specificity. The entire search process was documented by following the PRISMA guidelines [15]. We used medRxiv as a repository for gray literature data.

### Search strategies

Following the research question and the databases, we performed a search using MeSH, Emtree, and DECs terms, as well as title and abstract searches. For details on the search strategies, see **S2 File.**

### Study selection

All searches were downloaded in the RIS format and uploaded to the Zotero^®^ reference manager to create a database of the selected articles. First, we filtered out duplicate articles; subsequently, we downloaded the search elements and conducted a second filter in Microsoft Excel^®^ on the author names, titles, and DOI to identify the remaining repeated articles. After completing a review of duplicate articles, six authors trained in ocular inflammatory diseases were divided into three groups for a paired review of the remaining articles.

**Fig 1:**
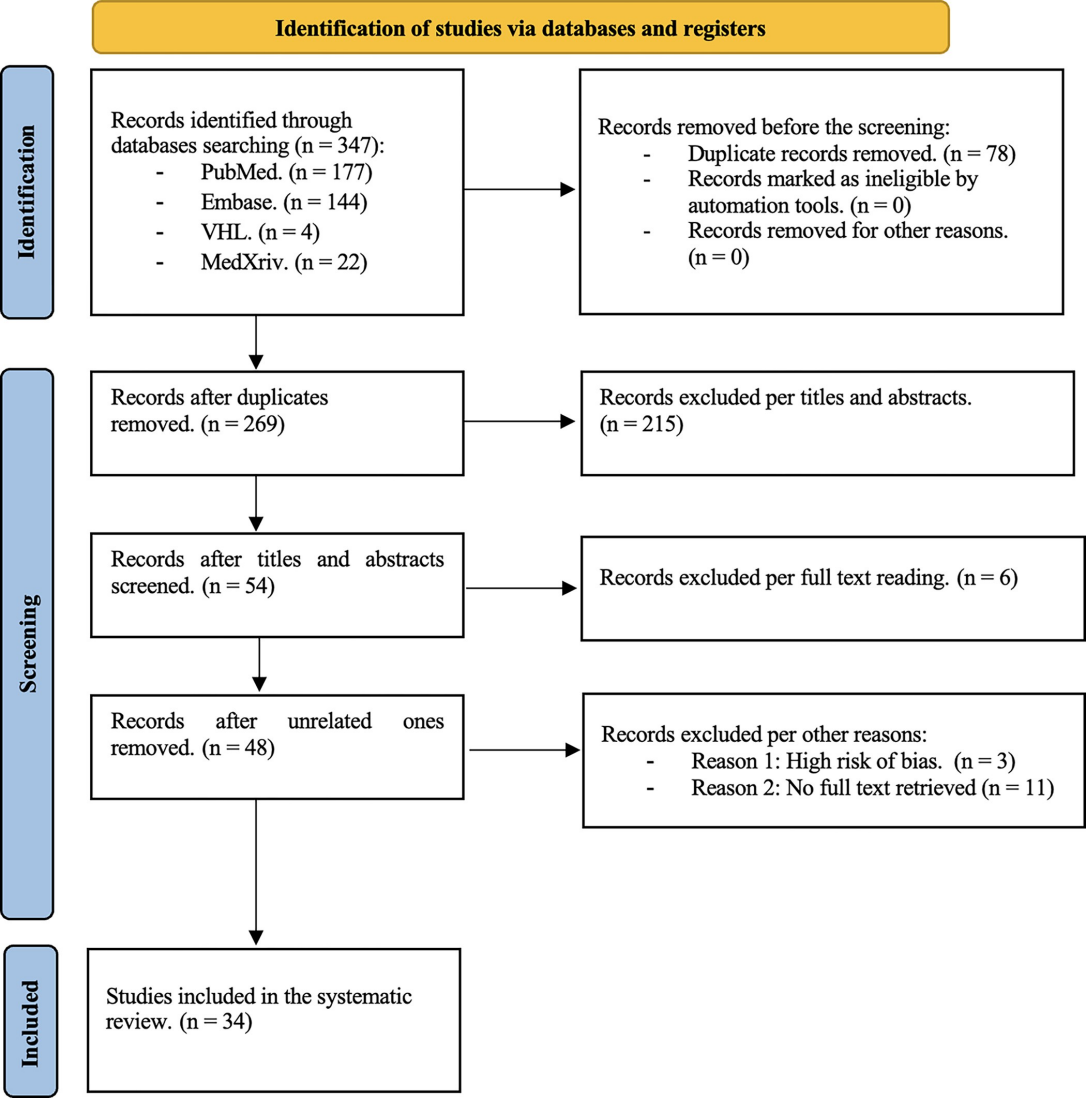
PRISMA flow diagram.

In the paired reviews, first, the titles and abstracts and then the full-text articles were reviewed for selection against the inclusion and exclusion criteria, following which the articles were labeled as included, excluded, or in doubt in a Microsoft Excel^®^ database. Disagreement during the paired review resulted in all authors reviewing an article again until a consensus was reached. In cases where disagreements remained, the final judgment was made by consulting two uveitis specialists. In this step, the level of agreement was 87% between the groups (Group 1: 90%; Group 2: 84.4%; and Group 3: 90%) **(Fig 1).**

### Data extraction

The selected articles were coded and downloaded using the assigned code for extracting information. In this step, articles retrieved without a full text were excluded. The papers were divided among the six trained reviewers, who rechecked that the articles met the inclusion criteria. Subsequently, the information was extracted as follows: article code, author, article title, year, DOI, design, age, uveitis location, etiologic diagnosis, descriptive diagnosis, course, activity, total population, population with surgery, control population, ECD, CV, HEX, CCT, cataract, IOP, need for keratoplasty, and relevant corneal findings in patients with ACI and controls.

### Assessment of methodological quality

Two authors assessed methodological quality using the tools provided by Hassan Murad et al. [16] for the case series. This tool evaluates the determination of exposure, whether other changes that explain the exposure were ruled out, whether the results were appropriately analyzed, the follow-up time, and, if sufficient, the reproducibility of the findings, including a description of the methods. In contrast, for cross-sectional studies, we used the validated tool of Hoy et al. [17]. This scale assesses bias by evaluating the sample’s representativeness, the type of sampling, response bias, data collection methods, case definition, the validity of measurements, duration of follow-up, prevalence measurements, and reproducibility of the study. Each item is scored, and the scores are summed for each study: a score of 0–3 indicates a high risk of bias, 4–6 indicates moderate risk and 7–10 indicates a low risk of bias.

Finally, to evaluate the cohort and case-control studies, we used the McMaster University group’s scale Clinical Advances Through Research and Information Translation (CLARITY) study (10). For case-control studies, this tool evaluates adequate exposure, case ascertainment safety, control selection, and matching. For cohort studies, it evaluates the selection of the exposed and unexposed cohorts, exposure, temporality of outcomes, group matching, reliability of outcomes, prognostic assessment, outcome analysis, follow-up, and reporting of possible cointerventions. In this case, the assessment items were scored as definitely yes (low risk of bias), probably yes, probably not, and definitely no (high risk of bias).

Although these tools did not explicitly determine the inclusion or exclusion of a study, for this research, a study was excluded if both coauthors who reviewed the risk of bias assessment considered that study to be at high risk of bias.

### Data processing and analysis techniques

To synthesize information, one author extracted the data from the selected articles. Initially, the data were synthesized in validated tables with all characteristics of the studies. Subsequently, a narrative synthesis of the relevant findings was performed following the PRISMA guidelines [15]. This process was repeated to summarize the effects of IOP and cataract surgery on endothelium and corneal thickness in patients with ACI and the need for keratoplasty. However, it was impossible to meta-analyze in this case because the reported data was not uniform across studies. Finally, a second author rectified the information presented in the tables.

For data analysis, we used data pertaining to the eyes and not to the patient. Cases were defined as eyes with ACI, and controls were defined as healthy eyes that could be from independent controls or, in some articles, as contralateral eyes. In our study, because the measurements were continuous quantitative (ECD, CV, HEX, and CCT), they were analyzed using standardized mean differences. Because the authors from the different studies had used different measuring equipment and standards, we expected high baseline heterogeneity, and thus, the use of random effects was preferred in the meta-analyses. Forest plots were performed using Review Manager (RevMan 5.4; The Nordic Cochrane Center, The Cochrane Collection, Copenhagen, Denmark). Furthermore, a subgroup analysis was performed for the ACI’s activity degree and course. All studies that provided these data were included.

We defined the activity and course of disease following the Standardization of Uveitis Nomenclature (SUN) recommendations. [18] We characterized uveitis that has been treated and currently shows no inflammation, as inactive, as evidenced by Grade 0 + cells in the anterior chamber when possible. Furthermore, if an article did not report the specific inflammation grade, uveitis was deemed inactive if, in the article’s methods, the group was referred to as “inactive/no inflammation.” Active uveitis referred to any inflammation grade >0 + cells necessitating treatment.

Acute uveitis is characterized by a sudden onset and limited duration. Because the time between recurrences was not distinctly defined in some studies, we analyzed recurrent and chronic uveitis in the same group to assess the effects of recurrences and chronicity on endothelial parameters.

Subsequently, sensitivity analyses were performed on the articles, excluding articles with low methodological quality determined by the risk of bias. Moreover, the quality of data collection and measurement in the studies with extreme values were reviewed to decide whether to include or exclude them from the meta-analysis. Heterogeneity analysis was evaluated using the I^2^ statistic, Cochran’s Q, and Tau. The cutoff points for heterogeneity in the meta-analysis were 0%–30% low, 31%–50% moderate, and 51%–90% high. In funnel plots with >10 studies, we used symmetry and Egger’s test to determine publication bias. This analysis used the Jamovi 2.3 Software [19–21].

## Results

### Study selection and characteristics

The initial search yielded 347 studies (Pubmed 177, Embase 144, VHL 4, and medRxiv 22). After the studies were reviewed, 78 duplicate ones were removed. 215 studies were removed during the paired review phase of abstract and title and 6 during the full-text review because they did not meet the inclusion criteria. Furthermore, 11 studies were discarded because the full text could not be retrieved; 3 studies were excluded due to a high risk of bias identified. In total, 34 studies were included in our systematic review; 25 were cross-sectional, 7 were case series, 1 was a cohort, and 1 was a case-control study. For the meta-analyses, the number of studies varied depending on data availability for each analysis.

The results of each analysis are presented below according to the number of studies available for each variable of interest. In total, 1,388 eyes with ACI were included, of which 583 eyes belonged to women, and 581 were from men, with a mean age of 36.6 (SD 9.8) years. Regarding the etiology of ACI, 532 (41. 9%) were idiopathic, 454 (35.7%) noninfectious, and 120 (9.4%) infectious; specific underlying etiologies of ACI were herpetic, Fuchs uveitis syndrome, Posner–Schlossman Syndrome, cytomegalovirus endothelitis, HLA B27+ anterior uveitis, uveitis associated with juvenile idiopathic arthritis, Bechet’s syndrome, toxoplasmosis, and Vogt–Koyanagi–Harada disease, among others **(Table 1)**.

**Table 1:** Characteristics of the corneal endothelium and central corneal thickness in the included studies.

Study authors (Year)	Type of study	Number of eyes with ACI (F/M)	Course n (%)	Etiology, n (%)	Mean ECD ± SD (cells/mm2)	Mean CV ± SD (%)	Mean HEX ± SD (%)	Mean CCT ± SD (μm)
Fung et al. (2021) [12]	Cross-sectional	99 (66/33)	ND	Idiopathic: 40 (40) Noninfectious: 58 (59) Infectious: 1(1)	3,510 ± 331.2	24.4 ± 2.5	71.9 ± 6.1	ND
Zina et al. (2021) [22]	Cross-sectional	38 (17/21)	ND	Idiopathic: 15 (39.4) Noninfectious: 17 (44.7) Infectious 6: (15.8)	2,642 ± 236	31 ± 5	64.7 ± 5.8	555 ± 44
Simsek et al. (2021) [23]	Cross-sectional	45 (24/21)	Chronic: 45 (100)	Idiopathic: 45 (100)	2,286.2 ± 283.4	37.7 ± 5.6	42.1 ± 5.3	539.5 ± 32.1
Sevinc et al. (2021) [9]	Cross-sectional	34 (19/15)	Acute: 34 (100)	Idiopathic: 18 (52.9) Noninfectious: 11 (32.3) Infectious: 5 (14.7)	2,607.7 ± 277.6	31.68 ± 8.16	63.85 ± 11.14	571.47 ± 55.99
Sravani et al. (2020) [5]	Cross-sectional	31 (9/22)	Chronic: 31 (100)	Idiopathic: 31 (100)	ND	ND	ND	511.5 ± 44.1
Alfawaz et al. (2016) [6]	Cross-sectional	56 (ND/ND)	Acute: 17 (38.5) Chronic: 35 (67.3)	Idiopathic: 21 (40.4) Noninfectious: 26 (50) Infectious: 5 (9.6)	2,351 ± 450.9	34.7 ± 8.0	52.3 ± 10.5	544.5 ± 39.6
Guclu et al. (2019) [7]	Cross-sectional	56 (27/29)	Chronic: 56 (100)	Idiopathic: 22 (39.2) Noninfectious: 34 (60.7)	2,540 ± 619	38 ± 29	49 ± 21	522 ± 39
Chen et al. (2021) [24]	Cross-sectional	140 (66/46)	ND	ND	2,843.1 ± 465.7	32.1 ± 6.4	59.8 ± 14.5	ND
Kam et al. (2021) [25]	Cross-sectional	38 (14/24)	Chronic: 38 (100)	Idiopathic: 23 (60.5) Infectious: 15 (39.5)	1,912 ± 564.5	46 ± 28.9	29 ± 9.6	ND
Cetin et al. (2022) [26]	Cross-sectional	55 (ND/ND)	ND	Idiopathic: 21 (72.4) Noninfectious: 8 (27.5)	2,971 ± 163	26.1 ± 4.2	67.7 ± 4.5	558.7 ± 27.8
Sen et al. (2018) [27]	Cross-sectional study	37 (14/23)	ND	Idiopathic: 21 (56.7) Noninfectious: 16 (43.2)	ND	ND	ND	550.7 ± 49.5
Cai et al. (2022) [28]	Cross-sectional	17 (6/11)	Chronic: 17 (100)	Idiopathic: 17 (100)	2,320.2 ± 329.	35.4 ± 6.8	56.5 ± 9.9	538.80 ± 37.01
Banaee et al. (2016) [29]	Case series	30 (12/18)	Acute: 30 (100)	Idiopathic: 30 (100)	2,787.5 ± 497.5	33.3 ± 4.5	ND	514.0 ± 23.1
Ghiță et al. (2019) [30]	Cohort	27 (11/16)	Acute: 27 (100)	ND	2,541 ± 351.21	34.07 ± 7.05	51.33 ± 17.19	562.40 ± 51.28
Cankaya et al. (2018) [31]	Cross-sectional	33 (15/18)	Chronic: 33 (100)	Noninfectious: 33 (100)	2,739 ± 164.18	32.9 ± 4.76	44.7 ± 6.51	545.75 ± 40.89
Ozdamar et al. (2010) [32]	Cross-sectional	69 (27/40)	ND	Noninfectious: 69 (100)	ND	ND	ND	G1: 584.7 ± 20.9 G2: 540.5 ± 36.1
Agra et al. (2014) [33]	Cross-sectional	24 (15/17)	Acute: 24 (100)	ND	ND	ND	ND	G1: 564.2 ± 44.2 G2: 529.5 ± 33.1
Sen et al. (2015) [34]	Cross-sectional	38 (17/21)	Chronic: 38 (100)	Idiopathic: 38 (100)	ND	ND	ND	548.8 ± 42.1
Heinz et al. (2012) [35]	Cross-sectional	30 (16/14)	Acute: 30 (100)	Idiopathic: 12 (40) Noninfectious: 16 (54) Infectious: 2 (7)	ND	ND	ND	Active: 645 ± 62 Inactive: 582 ±36
Szepessy et al. (2016) [13]	Cross-sectional	15 (8/7)	Chronic: 15 (100)	Idiopathic: 15 (100)	2,648.4 ± 121.44	38.5 ± 2.32	41 ± 3.97	543.5 ± 35.99
Pillai et al. (2015) [36]	Case series	13 (4/9)	Acute: 7 (53.84) Chronic: 3 (23.07) Recurrent: 3 (23.07)	Idiopathic: 12 (92.30) Infectious: 1 (7.69)	2,479.23 ± 175.78	ND	ND	ND
Dikmetas et al. (2022) [37]	Cross-sectional	64 (35/29)	Chronic: 30 (100)	Noninfectious: 30 (100)	2,124.9 ± 417.4	45.2	38.4	ND
Brooks et al. (1986) [38]	Case series	13 (10/3)	Acute: 4 (13.33) Chronic: 22 (73.33) Recurrent: 4 (13.33)	Idiopathic: 30 (100)	2,374.30 ± 786.31	ND	ND	ND
Ozer et al. (2019) [39]	Case series	21 (13/8)	Chronic: 21 (100)	Idiopathic: 21 (100)	2,228 ± 365	ND	ND	ND
Yilmaz et al. (2022) [40]	Case–control	30 (22/8)	Chronic: 30 (100)	Noninfectious: 30 (100)	3,335 ± 329	33 ± 5	58 ± 13	572 ± 27
Olsen et al. (1981) [41]	Case series	15 (10/5)	Acute: 6 (40) Recurrent: 9 (60)	Idiopathic: 15 (100)	2,709 ± 243	ND	ND	ND
Vannas et al. (1983) [10]	Case series	15 (5/10)	Acute: 10 (66.6) Recurrent: 5 (33.3)	Infectious: 15 (100)	2,487 ± 358	ND	ND	ND
Cankaya et al. (2014) [8]	Cross-sectional	40 (ND/ND)	Acute: 20 (50) Recurrent: 20 (50)	Noninfectious: 40 (100)	ND	ND	ND	Acute: 595.50 ± 39.5 Recurrent: 528.35 ± 19.1
Mocan et al. (2011) [42]	Cross-sectional	40 (16/24)	Chronic: 40 (100)	Idiopathic: 40 (100)	2,545 ± 234	39 ± 6	44.1 ± 6.2	ND
Setälä et al. (1979) [43]	Cross-sectional	60 (32/28)	Acute: 17 (28.3) Chronic 14 (23.3) Recurrent: 29 (48.3)	Idiopathic: 45 (75) Noninfectious: 15 (25)	2,632 ± 405	ND	ND	ND
Turan et al. (2012) [44]	Cross-sectional	85 (ND/ND)	Recurrent: 51 (100)	No Infectious: 51 (100)	ND	ND	ND	544.65 ± 17.91
Alanko et al. (1986) [45]	Case series	14 (8/6)	Chronic: 14 (100)	Infectious: 14 (100)	2,724 ± 631	27.4 ± 6.5	2.1 ± 1.1	ND
Reijo et al. (1983) [11]	Case series	14 (8/6)	Acute: 14 (100)	Infectious: 14 (100)	2,496 ± 409	ND	ND	ND
Choi et al. (2016) [46]	Cross-sectional	42 (13/29)	Chronic: 17 (40.5) Recurrent: 4 (9.5) ND: 21 (50)	Infectious: 42 (100)	ND	ND	ND	ND

ACI: Anterior chamber inflammation, F: Female, M: Male, ND: No data reported, ECD: Endothelial cell density, CV: Coefficient of variation, HEX: Hexagonality, CCT: Central corneal thickness, G1: Group 1, G2: Group 2.

### Risk of bias within the selected articles

Different tools were applied to evaluate the studies according to the design of each one. Following Hassan Murad et al.’s criteria [16], we included all eight case reports and case series that we found, although 1 of them had lower quality than the others because we could not adequately determine the exposure within the study. In contrast, the 25 cross-sectional studies were evaluated with the tool of. Using Hoy et al.’s criteria [17], we identified a low risk of bias in all 25 cross-sectional studies, although we scored 2 of these studies as 7/10; in one study, the sample was not an accurate representation of the target population and in the other cases were not defined. None of the included studies detailed a random sampling method or indicated that a census was conducted. We determined that only 1 study’s sample closely represented the national population in terms of relevant variables, such as age and gender.

We rated the case-control study as low risk according to the five items on the CLARITY tool [47]. Lastly, we also used the eight CLARITY items to evaluate the included cohort study [47] and found that the study lacked any exposure evaluation. Furthermore, the authors of the study could not ascertain that the outcome of interest was absent at the beginning, and we could not confidently assess the presence or absence of prognostic factors. Nonetheless, we included this study based on the score and the recommendation of the evaluation tool **(S3 File).**

### Endothelial parameters and CCT in eyes with ACI

#### Analysis of ECD

**Fig 2:**
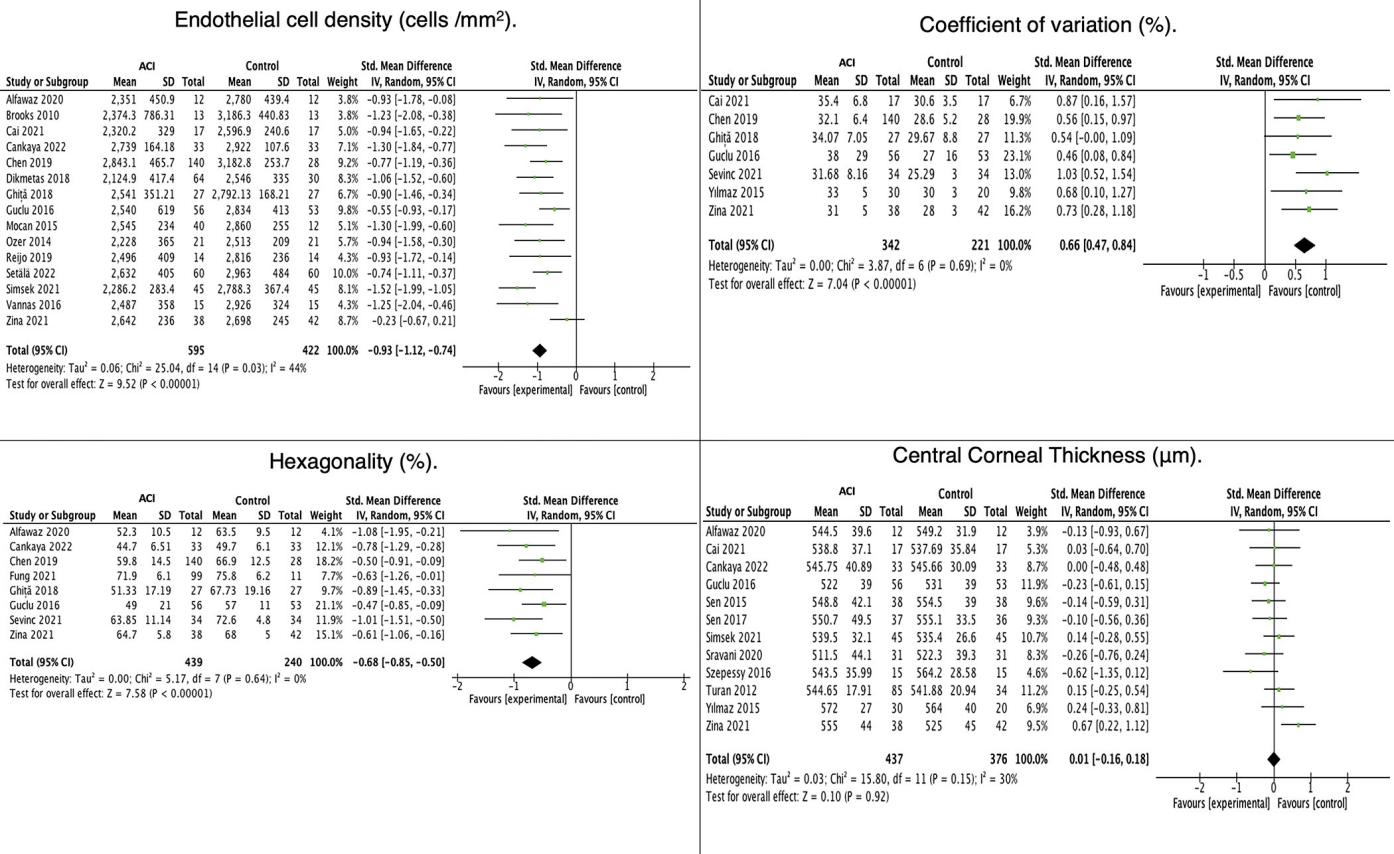
Effects of uveitis on morphological endothelial parameters.

**Fig 3:**
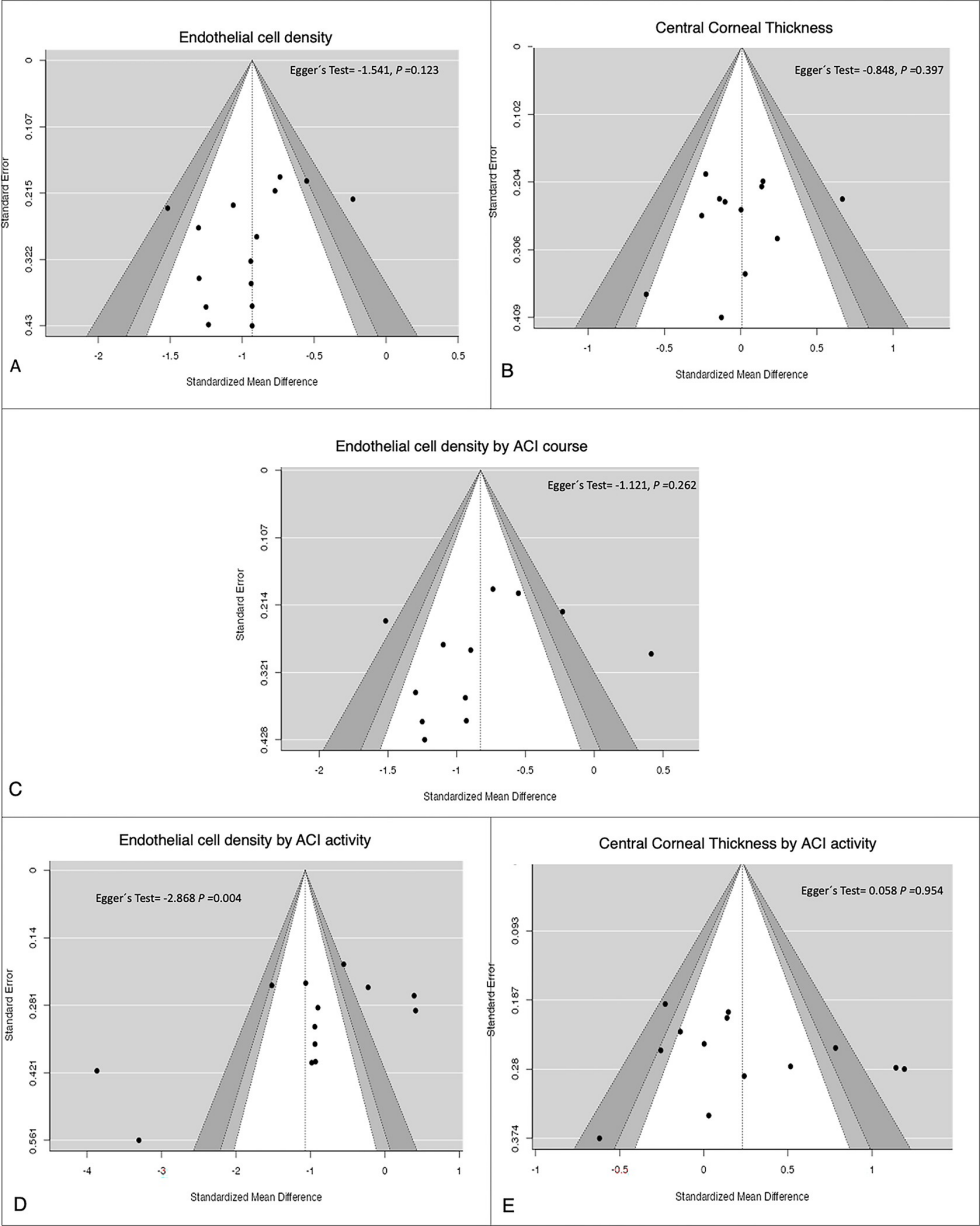
Funnel plots.

For the ECD analysis, 22 studies with 1,426 eyes were included; however, during the application of the sensitivity analysis, the number of studies was reduced to 15 and 1,017 eyes **(S4 File and Fig 2).** In the meta-analysis, we found a statistically significant difference between the ACI group and the control group with a standardized mean difference (SMD) = −0.93 (95% confidence interval [CI]: −1.12, −0.74) (P ≤ 0.001); I^2^ 44% (P = 0.03). The funnel plot had an approximately symmetrical distribution in the publication bias analysis, and Egger’s test = −1,542 (P = 0.123) **(Fig 3).**

#### Analysis of CV

Eight studies (679 eyes) were included originally, but one was excluded following sensitivity analysis, leaving 563 eyes **(S4 File and Fig 2).** In the meta-analysis, we observed that SMD = 0.66 (95% CI 0.47, 0.84) (P < 0.001); I^2^ 0% (P = 0.69) **(Fig 2). Fig 2** shows the results of comparing endothelial parameters (ECD, CV, and HEX) and CCT between eyes with ACI and healthy controls. **Fig 3** shows the funnel plots of the publication bias analyses performed on meta-analyses with >10 studies.

**Fig 3A, 3B, 3C and 3E** show the plots for the meta-analysis of overall ACI-ECD, overall ACI-CCT, and ECD when comparing acute vs. chronic-recurrent ACI and CCT comparing active vs. inactive ACI. They show an approximately symmetrical distribution, and Egger’s test is not significant for publication bias. **Fig 3D** shows the meta-analysis of ECD when comparing active vs. inactive ACI, showing a nonsymmetrical distribution in the funnel plot and Egger’s test with statistical significance demonstrating publication bias.

#### Hexagonality

Fourteen studies (963 eyes) were included, which was reduced to eight (679 eyes) following the sensitivity analysis **(S4 File and Fig 2).** In the meta-analysis, we observed that SMD = −0.68 (95% CI: −0.85, −0.50) (P < 0.001); I^2^ 0% (P = 0.64) **(Fig 2).**

#### Analysis of CCT

In the meta-analysis of CCT, 15 included studies contained 995 eyes in total. However, the sensitivity analysis reduced the number of studies to 12 and 813 eyes **(S4 File and Fig 2).** The meta-analysis showed no statistically significant difference between the ACI and the control group (SMD = 0.01; 95% CI: −0.16, 0.18; P = 0.92; I^2^ = 30% [P = 0.15]). The funnel plot showed an approximately symmetrical distribution in the publication bias analysis, and Egger’s test = −0.848 (P = 0.397) **(Fig 3).**

### Effects of inflammatory activity on endothelial parameters and corneal thickness

#### Analysis of ECD

**Fig 4:**
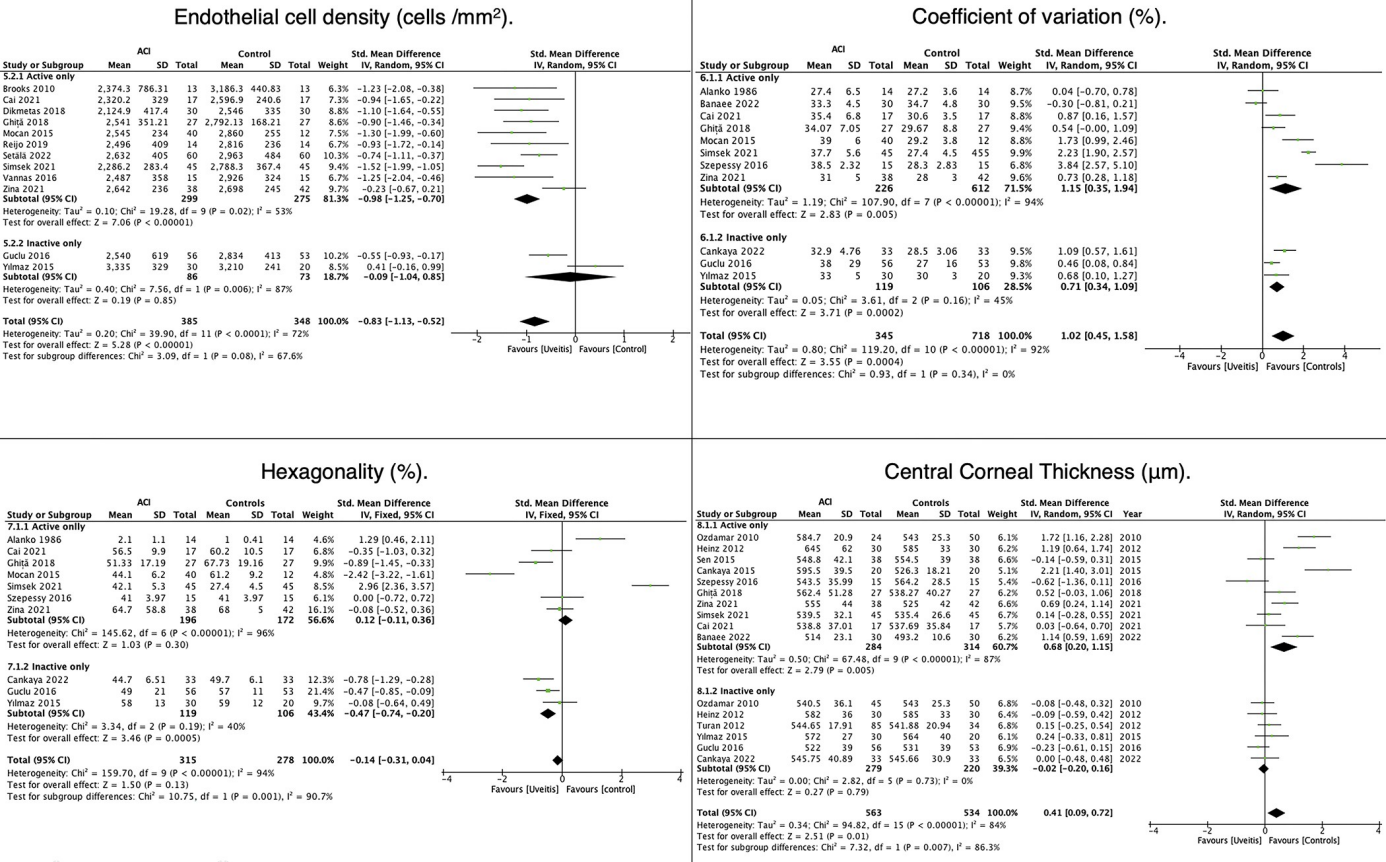
Effects of inflammatory activity on endothelial parameters and central corneal thickness.

In the meta-analysis of ECD, 13 studies with 799 eyes were included; after the sensitivity analysis, there were 12 studies with 733 eyes **(S4 File and Fig 4).** Subgroup analysis showed that eyes with active ACI had fewer cells/mm^2^ than healthy controls (SMD = −0.98; 95% CI: −1.25, −0.70; P < 0.001), and according to the Q test, there was moderate heterogeneity in the results (Q = 19.28, P = 0.02, Tau^2^ = 0.10, I^2^ = 53%) **(Fig 4).** Conversely, there was no statistically significant difference between inactive ACI cases and healthy controls (SMD = −0.09; 95% CI: −1.04, 0.85; P = 0.85). However, it should be noted that this group showed high heterogeneity (Q = 7.56, P = 0.006, Tau^2^ = 0.40, I^2^ = 87%) **(Fig 4).** The subgroup analysis showed no statistically significant difference between active and inactive ACI (P = 0.08) **(Fig 4).** In the publication bias analysis, we observed a nonsymmetrical distribution in the funnel plot and Egger’s test = −2,868 (P = 0.004) **(Fig 3). Fig 4** presents the findings for comparing the endothelial parameters (ECD, CV, and HEX) and CCT in eyes with ACI regarding inflammatory activity.

#### Analysis of CV

Subgroup analysis showed that active ACI had a significantly higher CV than healthy controls (SMD = 1.15; 95% CI: 0.35, 1.94; P = 0.005). The Q statistic showed high heterogeneity (Q = 107.9, P < 0.001, Tau^2^ = 1.19, I^2^ = 94%) **(Fig 4).** Similarly, in patients with inactive ACI, the CV was statistically significantly higher than the controls’ (SMD = 0.71; 95% CI: 0.34, 1.09; P = 0.0002), although we noted that this group presented moderate heterogeneity (Q = 3.61, P = 0.16, Tau^2^ = 0.05, I^2^ = 45%) **(Fig 4).** The subgroup analysis revealed no statistically significant difference between active vs. inactive ACI (P = 0.34) **(Fig 4).**

#### Hexagonality

There was no statistically significant difference in HEX for eyes with active ACI from that for healthy controls (SMD = 0.12; 95% CI: −0.11, 0.36; P = 0.30). However, this analysis revealed high heterogeneity (Q = 145.62, P < 0.001, I^2^ = 96%) **(Fig 4).** In contrast, eyes with inactive ACI displayed a significantly lower HEX than that of healthy controls (SMD = −0.47; 95% CI: −0.74, −0.20; P = 0.0005), with moderate heterogeneity (Q = 3.34, P = 0.19, I^2^ = 40%) **(Fig 4).** Subgroup analysis highlighted a significant difference between active and inactive ACI (P = 0.001) **(Fig 4).**

#### Analysis of CCT

Active ACI had significantly higher CCT than that of healthy controls (SMD = 0.68; 95% CI: 0.20, −1.15; P = 0.005), and heterogeneity was high (Q = 67.48, P < 0.00001, Tau^2^ = 0.50, I^2^ = 87%) **(Fig 4).** Inactive ACI did not evidence statistically significant differences versus healthy controls, SMD = −0.02 (95% CI: −0.20, 0.16) (P = 0.79), and heterogeneity was low (Q = 2.82, P = 0.73, Tau^2^ = 0.00, I^2^ = 0%) **(Fig 4).** Subgroup analysis indicated statistically significant differences between active and inactive ACI (P = 0.007) (**Fig 4**). The funnel plot of the publication bias analysis findings showed a symmetrical distribution and Egger’s test = 0.058 (P = 0.954) **(Fig 3).**

### Effects of ACI course on endothelial parameters and corneal thickness

#### Analysis of ECD

**Fig 5:**
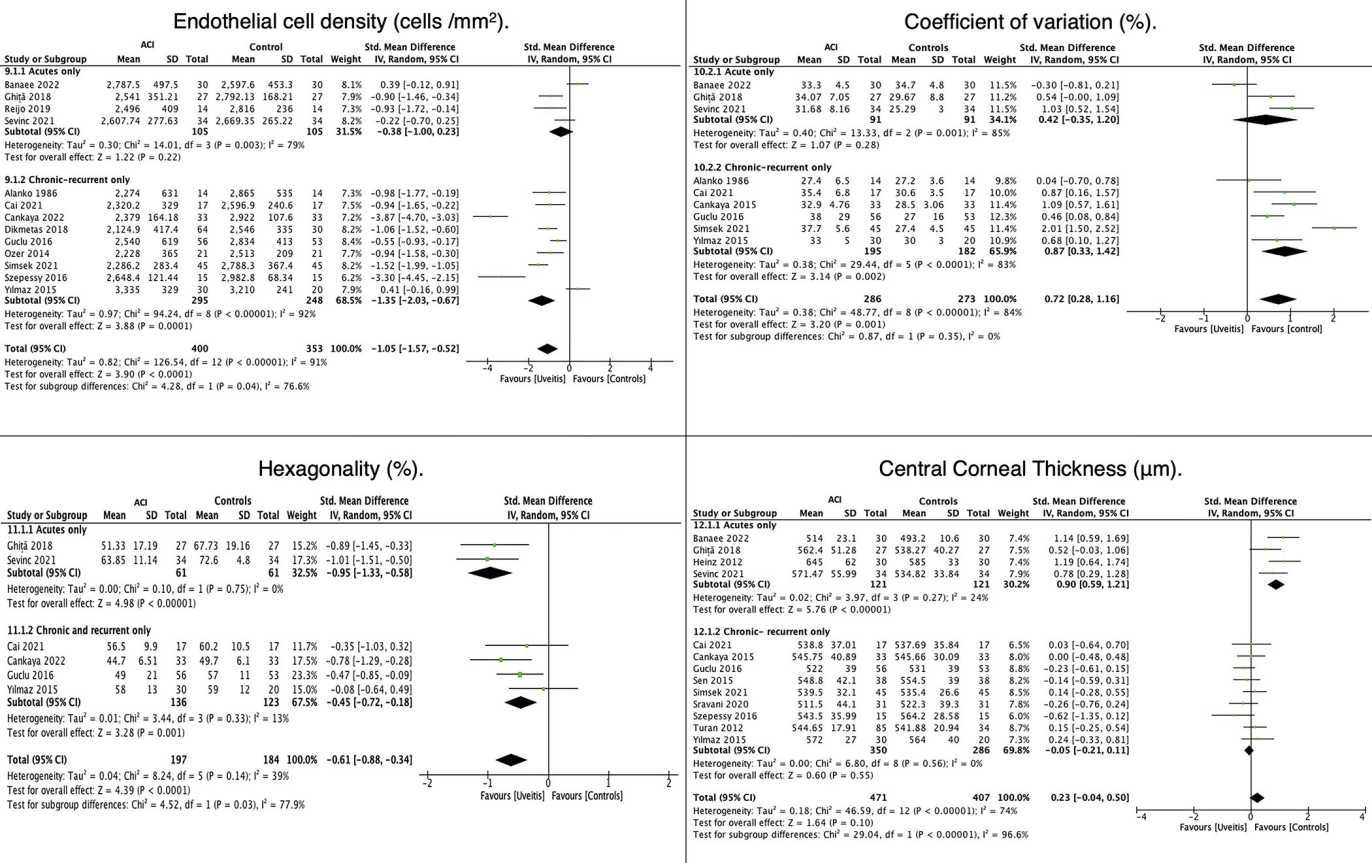
Effects of ACI course on endothelial parameters and corneal thickness.

In the comparison of ECD, there was no difference between acute ACI and healthy controls (SMD = −0.38; 95% CI: −1.0, 0.23; P = 0.22). However, there was a statistically significant difference when comparing the chronic-recurrent group with healthy controls (SMD = −1.35; (95% CI: −2.03, −0.67; P < 0.001) **(Fig 5).** The difference between subgroups was significant (P = 0.04), and there was high heterogeneity in both acute (Q = 14.01, P = 0.003, Tau^2^ = 0.30, I^2^ = 79%) and chronic-recurrent ACI (Q = 94.24, P < 0.00001, Tau^2^ = 0.97, I^2^ = 92%). The funnel plot showed a symmetrical distribution in the publication bias analysis, and Egger’s test = −1.121 (P = 0.262) **(Fig 3). Fig 5** shows the results for comparing endothelial parameters (ECD, CV, and HEX) and CCT in eyes with ACI regarding the course of ACI.

#### Analysis of CV

During the meta-analysis of CV, 10 studies were included, comprising a total of 589 eyes; this number reduced to 9 studies following the sensitivity analysis, and 559 eyes were evaluated **(S4 File and Fig 5).** When comparing acute ACI and healthy controls, there were no significant differences (P = 0.28), and heterogeneity was high (Q = 13.33, P = 0.001, Tau^2^ = 0.40, I^2^ = 85%) **(Fig 5).** However, a statistically significant difference was evident between chronic-recurrent uveitis and healthy controls (SMD = 0.87; (95% CI: 0.33, 1.42; P = 0.002), with high heterogeneity (Q = 29.44, P < 0.001, Tau^2^ = 0.38, I^2^ = 83%). Subgroup analysis of the CV showed no differences between groups (P = 0.35).

#### Hexagonality

For hexagonality, all courses of ACI showed differences from the controls: acute ACI, SMD = −0.95 (95% CI: −1.33, −0.58; P < 0.001) with low heterogeneity (Q = 0.10, P = 0.75, Tau^2^ = 0.00, I^2^ = 0%); chronic-recurrent ACI, SMD = −0.45 (95% CI: −0.88, −0.34; P = 0.001) with low heterogeneity (Q = 3.44, P = 0.33, Tau^2^ = 0.01, I^2^ = 13%). In the subgroup analysis, acute ACI showed less hexagonality than chronic or recurrent ACI (P = 0.03) **(Fig 5).**

#### Analysis of CCT

Acute ACI presented increased CCT than healthy controls (SMD = 0.90; 95% CI: 0.59, 1.21; (P < 0.001). However, there was no significant difference between chronic-recurrent ACI and healthy controls (SMD = −0.05; 95% CI: −0.21, 0.11; P = 0.55). The comparison of CCT between subgroups showed statistically significant differences (P < 0.001) (**Fig 5).**

### Effects of intraocular pressure and uveitic cataract surgery on endothelial and corneal thickness parameters

We identified a total of 20 articles describing IOP **(Table 2).** These studies provided information on IOP variation in relation to different conditions or interventions; however, they did not report their relationship with endothelial parameters or corneal thickness. Simsek et al. observed that IOP was significantly higher in eyes with ACI (17.7 ± 3.6 mmHg) than in the fellow eyes (14.8 ± 3.2 mmHg) and in the healthy group (14.2 ± 2.3 mmHg) (P ≤ 0.05), indicating that IOP is higher in the presence of ACI than in eyes without this condition [23]. In contrast, findings from numerous studies indicated no statistically significant differences in IOP between the ACI groups and other groups [29,39].

**Table 2:** Intraocular pressure and endothelial structural and morphologic parameters.

Study authors (Year)	IOP in ACI group	IOP in controls (without ACI)	Mean ECD ± SD (cells/mm2)	Mean CV ± SD (%)	Mean HEX ± SD (%)	Mean CCT ± SD (μm)
Fung et al. (2021) [12]	19.4 ± 5.5	ND	3,510 ± 331.2	24.4 ± 2.5	71.9 ± 6.1	ND
Zina et al. (2021) [22]	12 ± 1,4	ND	2,642 ± 236	31 ± 5	64.7 ± 5.8	555 ± 44
Simsek et al. (2021) [23]	17.7 ± 3.6	14.2 ± 2.3	2,286.2 ± 283.4	37.7 ± 5.6	42.1 ± 5.3	539.5 ± 32.1
Sevinc et al. (2021) [9]	15.71 ± 7.06	15.91 ± 2.64	2,607.74 ± 277.63	31.68 ± 8.16	63.85 ± 11.14	571.47 ± 55.99
Sravani et al. (2020) [5]	14 (IQR 12–16)	ND	ND	ND	ND	511.5 ± 44.1
Alfawaz et al. (2016) [6]	19.7 ± 6.6	ND	2,351 ± 450.9	34.7 ± 8.0	52.3 ± 10.5	544.5 ± 39.6
Guclu et al. (2019) [7]	14.4 ± 2.7	14.0 ± 2.8	2,540 ± 619	38 ± 29	49 ± 21	522 ± 39
Cetin et al. (2022) [26]	14.7 ± 4.2	16.8 ± 2.6	2,971 ± 163	26.1 ± 4.2	67.7 ± 4.5	558.7 ± 27.8
Sen et al. (2018) [27]	12.9 ± 3.3	13.9 ± 3.1	ND	ND	ND	550.7 ± 49.5
Cai et al. (2022) [28]	16.8 ± 3.3	17.4 ± 2.7	2,320.2 ± 329.	35.4 ± 6.8	56.5 ± 9.9	538.80 ± 37.01
Banaee et al. (2016) [29]	12 ± 1.7	12 ± 1.7	2,787.5 ± 497.5	33.3 ± 4.5	ND	514.0 ± 23.1
Ozdamar et al. (2010) [32]	G1: 13.1 ± 1 G2: 12.6 ± 3.4	14.3 ± 3.6	ND	ND	ND	G1: 584.7 ± 20.9 G2: 540.5 ± 36.1
Agra et al. (2014) [33]	G1: 10.8 ± 4.5 G2: 12.27 ± 3	ND	ND	ND	ND	G1: 564.2 ± 44.2 G2: 529.50 ± 33.1
Sen et al. (2015) [34]	13.1 ± 4.3	14.6 ± 3.4	ND	ND	ND	548.8 ± 42.1
Heinz et al. (2012) [35]	G1: 14.3 ± 5.8 G2: 17 ± 6.2	G1: 17.6 ± 4.3 G2: 17.3 ± 2.9	ND	ND	ND	Active: 645 ± 62 Inactive: 582 ± 36
Szepessy et al. (2016) [13]	15.7 ± 2.05	13.3 ± 1.33	2,648.4 ± 121.44	38.5 ± 2.32	41 ± 3.97	543.5 ± 35.99
Ozer et al. (2019) [39]	15 ± 2	15 ± 2	2,228 ± 365	ND	ND	ND
Cankaya et al. (2014) [8]	G1: 19.87 ± 2.92 G2: 15.89 ± 2.58	5.59 ± 2.74	ND	ND	ND	Acute: 595.50 ± 39.5 Recurrent: 528.35 ± 19.1
Setälä et al. (1979) [43]	12.3	15	2,632 ± 405	ND	ND	ND
Turan et al. (2012) [44]	13.58 ±1.66	13.13 ± 1.77	ND	ND	ND	544.65 ± 17.91

*Notes*: ND: No data reported, IOP: Intraocular pressure, ECD: Endothelial cell density, CV: coefficient of variation, HEX: hexagonality, CCT: central corneal thickness, G1: Group 1, G2: Group 2.

With respect to endothelial parameters, Guclu et al. and Alfawaz et al. found associations between IOP and endothelial cell parameters in patients with uveitis [6,7]. Guclu et al. observed that increased IOP during active uveitis was associated with a higher CV and decreased ECD [7], the latter of which was also described by Alfawaz et al. [6]. These findings suggest that high IOP is associated with impaired endothelial cell health in patients with uveitis. Furthermore, some studies reported associations between greater CCT and ACI [28,33].

There are limited studies addressing the relationship between surgical procedures and endothelial cell parameters in patients with ACI. Some have identified surgery as the sole factor contributing to endothelial cell loss [5,12,13]. However, while noting a decrease in ECD, other studies found no discernible differences between patients who had undergone surgical procedures (including glaucoma and cataract surgeries) and those who did not [42].

### Effects of ACI etiology on endothelial parameters and central corneal thickness

Although authors have reported endothelial damage for infectious and noninfectious uveitis, we could not quantitatively assess endothelial alterations and CCT differences between etiologies. For infectious causes, Vannas et al. presented a case series of 15 patients with herpetic keratouveitis and found pronounced pleomorphism and reduced ECD (15%) in patients experiencing severe acute active episodes compared to those with milder cases (1.4%). The authors identified elevated IOP in severe cases as the primary cause of this damage [10]. Similarly, examination of another case series of 14 patients with herpes zoster keratouveitis revealed that those with elevated IOP exhibited a lower ECD than those with normal IOP (20.2% vs. 15.3%) [11].

Furthermore, researchers have discussed the influence of noninfectious ACI on IOP; they found heightened endothelial damage and increased CCT during acute stages with elevated IOP [8]. Furthermore, Cankaya et al. in 2018 found a higher CV and lower HEX and ECD in patients with Behçet’s disease with previous inflammatory episodes in the anterior chamber than in unaffected individuals, emphasizing the negative impact of recurrent episodes on endothelial vitality [31]. An explanation of the effects of IOP and recurrences of ACI episodes was provided above.

## Discussion

Several studies have examined how ACI affects corneal endothelial parameters and CCT. Some of the studies indicate that the presence of ACI is associated with significantly low ECD and HEX and high CV and CCT [5,22,24]; however, other studies have found no such differences [10,41,45]. The wide variations in the findings and the lack of information underscore the importance of this systematic review and meta-analysis for clarifying the effects of ACI on the endothelium and CCT.

Eyes with ACI exhibited significantly decreased ECD, elevated CV, and reduced HEX compared with healthy controls. There are multiple possible reasons ACI is associated with endothelial damage, including direct damage from inflammatory cells and proteins in the aqueous humor. Interestingly, Alfawaz et al. found that higher flare levels in the anterior chamber (a reflection of increased protein levels) were significantly associated with lower ECD but not anterior chamber cellularity [6].

We found that active ACI had lower ECD and higher CV, whereas inactive ACI was only associated with higher CV and lower HEX compared to healthy controls. These two findings indicate that endothelial cells experience stress during active and inactive ACI, as CV is the most sensitive measure of corneal endothelial dysfunction [48]. During acute ACI episodes, when corneal edema is more evident, CV increases and ECD decreases because of water influx into the cell cytoplasm while HEX remains consistent; conversely, in inactive stages, HEX diminishes. In fact, HEX is a good indicator of healing progression after endothelial damage [48].

Considering that human endothelial cells *in vivo* exhibit no mitotic activity [2], the observed lower ECD during the active phase but not the inactive phase might be attributable to the limited number of studies exclusively focusing on patients with inactive ACI coupled with the high heterogeneity (>87%) rather than to actual endothelial cell regeneration. Longitudinal studies with extended follow-ups exploring endothelial parameters during inactive periods are crucial for further clarifying this matter.

Another leading cause of endothelial damage in patients with ACI is the long-term use of corticosteroids (topical or systemic) [49,50], which can lead to common complications such as cataracts and glaucoma [51]. The adverse effects of elevated IOP on the endothelium are well established, with both open-angle and acute angle-closure glaucoma reporting endothelial damage [43,46,52,53]. The duration of elevated IOP has been associated with this damage, as normal tension glaucoma has normal ECD [46].

Although we could not investigate the durations of elevated IOP, we believe that IOP plays an essential role in endothelial damage in hypertensive uveitis, such as herpetic uveitis. Therefore, IOP control should be one goal in treating patients with uveitis, not only to minimize the possible glaucomatous damage [54] but also because of the importance of the corneal endothelium.

Regarding intraocular surgery, the data are contradictory. Some research suggests surgery is the primary cause of endothelial cell loss in patients with ACI [6,12,13]; however, some researchers have found decreased ECD and HEX and increased CV in patients with ACI who have not undergone surgery [42]. More studies are needed to determine the heightened risk of endothelial damage in patients with ACI with ocular surgery vs. those without surgical procedures.

Subanalysis regarding the course of ACI identified that acute episodes of ACI did not show any differences in ECD and CV compared with controls, although HEX was lower. However, this was based on two studies only. In contrast, chronic-recurrent uveitis showed significant differences in ECD, CV, and HEX. Although we could not measure the duration of uveitis, we attribute this finding to chronic exposure to inflammation. Some researchers have reported lower ECD and HEX and higher CV in eyes with Fuchs uveitis syndrome (a chronic low-grade unilateral uveitis) than in the contralateral eyes or in healthy controls [5,23,34].

We found that patients with active and acute ACI displayed increased CCT than the healthy control eyes or those with inactive or chronic-recurrent ACI. This could be attributed to the rise in IOP during acute ACI episodes, which affects endothelial permeability [23]. This variation in CCT poses potential inaccuracies when measuring IOP using applanation tonometers during acute ACI episodes [39]. Utilizing noncontact measuring instruments could provide more accurate estimates of IOP in patients active and acute ACI [55]. However, such equipment has not been comprehensively tested in patients with ACI, and their use requires further investigation [56].

Similarly to increased CCT in ACI, a thickening of the nerve fiber layer has been noted in cases of acute posterior uveitis. This is often succeeded by a thinning during inactivity, emphasizing the need for regular monitoring. [57,58] Consequently, pachymetry assessments in patients with ACI could be beneficial in screening for corneal microedema. The latter can chronically influence corneal clarity and endothelial metrics (ECD, HEX, and CV) [59] and increase the risk of keratoplasty for patients with ACI [14].

Our analysis has some limitations. First, the authors of the selected studies used different devices to measure the endothelial variables; to counter this problem, we used SMD to pool changes in the continuous endothelial variable data across studies. Second, all studies included in the meta-analysis were observational, and such studies inherently carry a heightened risk of selection, confounding, or interpretation biases. To mitigate these concerns, we undertook a quality assessment and conducted a sensitivity analysis, omitting studies of lesser quality or with outlier values. Although this approach does not eliminate the biases of the included studies, it offers a more refined understanding of heterogeneity via more precise measures. Third, there was heterogeneity in disease activity status and course of the disease; this is explained by the limited number of studies in the subgroup analysis; therefore, we recommend expanding the available evidence in this field to confirm or reject our findings. Despite this limitation, however, we believe that our approach provides greater insight into the impacts of ACI at various times (active vs. inactive; acute vs. chronic-recurrent) on corneal endothelial parameters and improves the reliability of the study conclusions.

Fourth, the selected studies used different controls, specifically, the fellow eye or the eyes of healthy individuals. Though most researchers acknowledged this discrepancy in their studies, we considered it not especially consequential because intraclass correlation coefficient analysis verified the control group’s legitimacy. Finally, the ages of the patients varied widely, including pediatric patients. This could be seen as a bias; however, we considered it to be a strength because it allowed us to have a broader and more general perspective of the true effects of uveitis in all age groups.

## Conclusions

Patients with ACI typically exhibit reduced ECD and HEX and elevated CV and CCT; primary contributors to these changes are increased IOP, uveitis duration, and intraocular surgeries. Our results highlight potential inaccuracies in IOP measurements during acute ACI episodes and the potential necessity for monitoring the endothelial parameters and CCT in patients with chronic ACI. Further studies are essential for understanding the influence of etiology of ACI on the endothelium and evaluating the heightened risks associated with intraocular procedures in patients with ACI compared to those without.

## Supporting information

S1 File PRISMA checklist. (DOCX)

S2 File Search Strategy. (DOCX)

S3 File Risk of Bias. (DOCX)

S4 File Sensitivity analysis. (DOCX)
