# Supplementary material for: Quantitative changes in the corneal endothelium and central corneal thickness during anterior chamber inflammation: A systematic review and meta-analysis
Source: PLoS One. 2024 Jan 5;19(1):e0296784. doi: 10.1371/journal.pone.0296784 (PMC10769021; doi:10.1371/journal.pone.0296784)
Supplement: S2 File — (DOCX) [file pone.0296784.s002.docx]

**Supporting File 2: Search Strategy**

SEARCH STRATEGY PUBMED-DATE MARCH 08^TH^, 2023 7:31 PM

|  | **LINE** | **ARTICLES** |
| --- | --- | --- |
| **POPULATION** | ((((((uveitis[MeSH Terms]) OR (uveitis[Title/Abstract])) OR (uveitis, anterior[Title/Abstract])) OR (Iridocyclitis[MeSH Terms])) OR (Iridocyclitis[Title/Abstract])) OR (Iritis[MeSH Terms])) OR (Iritis[Title/Abstract]) | 42,617 |
| **EXPOUSURE** | (((((corneal thickness[Title/Abstract]) OR (Hexagonality[Title/Abstract])) OR (endothelial cell density[Title/Abstract])) OR (specular microscopy[Title/Abstract])) OR (corneal polymegathism[Title/Abstract])) OR (Corneal Edema[Title/Abstract]) | **13,249** |
| **SEARCH STRATEGY** | ("uveitis"[MeSH Terms] OR "uveitis"[Title/Abstract] OR "uveitis anterior"[Title/Abstract] OR "Iridocyclitis"[MeSH Terms] OR "Iridocyclitis"[Title/Abstract] OR "Iritis"[MeSH Terms] OR "Iritis"[Title/Abstract]) AND ("Cornea"[MeSH Terms] OR "Cornea"[Title/Abstract] OR "endothelium, corneal"[MeSH Terms] OR "endothelium corneal"[Title/Abstract]) AND ("corneal thickness"[Title/Abstract] OR "Hexagonality"[Title/Abstract] OR "endothelial cell density"[Title/Abstract] OR "specular microscopy"[Title/Abstract] OR (("Cornea"[MeSH Terms] OR "Cornea"[All Fields] OR "Corneal"[All Fields]) AND "polymegathism"[Title/Abstract]) OR "corneal edema"[Title/Abstract]) | **177** |

SEARCH STRATEGY EMBASE DATE MARCH 08^TH^, 2023 7:48PM

|  | **LINE** | **ARTICLES** |
| --- | --- | --- |
| **POPULATION** | 'uveitis'/exp OR uveitis:ab,ti OR 'iritis'/exp OR iritis:ab,ti | **72,954** |
| **EXPOUSURE** | 'corneal thickness':ab,ti OR hexagonality:ab,ti OR 'endothelial cell density':ab,ti OR 'specular microscopy':ab,ti OR 'corneal polymegathism':ab,ti OR 'cornea edema':ab,ti | **13,195** |
| **SEARCH STRATEGY** | ('uveitis'/exp OR uveitis:ab,ti OR 'iritis'/exp OR iritis:ab,ti) AND ('cornea'/exp OR cornea:ab,ti OR 'cornea endothelium'/exp OR 'cornea endothelium':ab,ti) AND ('corneal thickness':ab,ti OR hexagonality:ab,ti OR 'endothelial cell density':ab,ti OR 'specular microscopy':ab,ti OR 'corneal polymegathism':ab,ti OR 'cornea edema':ab,ti) | **144** |

SEARCH STRATEGY VHL DATE MARCH 08^TH^, 2023 7:57 PM

|  | **LINE** | **ARTICLES** |
| --- | --- | --- |
| **POPULATION** | Uveitis | **1711** |
| **EXPOUSURE** | Corneal thickness OR endothelial cell density OR specular microscopy OR corneal polymegathism OR Corneal Edema | **198** |
| **SEARCH STRATEGY** | Uveitis AND Cornea AND corneal thickness OR endothelial cell density OR specular microscopy OR corneal polymegathism OR Corneal Edema | **45** |

SEARCH STRATEGY MEDXRIV DATE MARCH 08^TH^, 2023 8:20 PM

|  | **LINE** | **ARTICLES** |
| --- | --- | --- |
| **POPULATION** | Uveitis | **110** |
| **INTERENTION** | Corneal endothelium | **22** |
| **SEARCH STRATEGY** | Uveitis AND Corneal endothelium | **22** |
