# Supplementary material for: Quantitative changes in the corneal endothelium and central corneal thickness during anterior chamber inflammation: A systematic review and meta-analysis
Source: PLoS One. 2024 Jan 5;19(1):e0296784. doi: 10.1371/journal.pone.0296784 (PMC10769021; doi:10.1371/journal.pone.0296784)
Supplement: S3 File — (DOCX) [file pone.0296784.s003.docx]

**Supporting Information 3: Risk of bias**

**S3 Table 1. Risk of bias or quality determination of case series.**

| Risk of bias of Case series and Case repots, Hassan Murad et al. | | | | | | | | | |
| --- | --- | --- | --- | --- | --- | --- | --- | --- | --- |
| Autor (año) | Selection | Ascertainment | | Causality | | | | Reporting | Final decision |
|  | 1 | 2 | 3 | 4 | 5 | 6 | 7 | 8 |  |
| Banaee, T. et al. (2016) | No | Yes | Yes | No | NA | NA | No | Yes | Include |
| Pillai, C. T et al. (2015) | No | Yes | Yes | No | NA | NA | No | Yes | Include |
| Brooks, A. M et al. (1986) | No | Yes | Yes | No | NA | NA | No | Yes | Include |
| Olsen, T. et al. (1981) | No | No | Yes | No | NA | NA | No | Yes | **Include Low quality** |
| Vannas, A et al. (1983) | No | Yes | Yes | No | NA | NA | No | Yes | Include |
| Alanko, H. I et al. (1986) | No | Yes | Yes | No | NA | NA | No | Yes | Include |
| Reijo, A. et al. (1983) | No | Yes | Yes | No | NA | NA | No | Yes | Include |

1. Does the patient(s) represent(s) the whole experience of the investigator (centre) or is the selection method unclear to the extent that other patients with similar presentation may not have been reported?
2. Was the exposure adequately ascertained?
3. Was the outcome adequately ascertained?
4. Were other alternative causes that may explain the observation ruled out?
5. Was there a challenge/rechallenge phenomenon?
6. Was there a dose–response effect?
7. Was follow-up long enough for outcomes to occur?
8. Is the case(s) described with sufficient details to allow other investigators to replicate the research or to allow practitioners make inferences related to their own practice?

**S3 Table 2. Risk of bias or cross-sectional quality determination**

| Autor (año) | 1 | 2 | 3. | 4 | 5 | 6 | 7 | 8 | 9 | 10 | Total | The risk is |
| --- | --- | --- | --- | --- | --- | --- | --- | --- | --- | --- | --- | --- |
| Fung, S. et al. (2021) | No | Yes | No | Yes | Yes | Yes | Yes | Yes | Yes | Yes | 8 | Low |
| Zina, S. et al. (2021) | No | Yes | No | Yes | Yes | Yes | Yes | Yes | Yes | Yes | 8 | Low |
| Simsek, M. et al. (2021) | No | Yes | No | Yes | Yes | Yes | Yes | Yes | Yes | Yes | 8 | Low |
| Sevinc, D. et al. (2021) | No | Yes | No | Yes | Yes | Yes | Yes | Yes | Yes | Yes | 8 | Low |
| Sravani, N. et al. (2020) | No | Yes | No | Yes | Yes | Yes | Yes | Yes | Yes | Yes | 8 | Low |
| Alfawaz, A. et al. (2016) | No | Yes | No | Yes | Yes | Yes | Yes | Yes | Yes | Yes | 8 | Low |
| Guclu, H. et al. (2019) | No | Yes | No | Yes | Yes | Yes | Yes | Yes | Yes | Yes | 8 | Low |
| Chen, Xu. et al. (2021) | No | Yes | No | Yes | Yes | Yes | Yes | Yes | Yes | Yes | 8 | Low |
| Kam, K. et al. (2021) | No | No | No | Yes | Yes | Yes | Yes | Yes | Yes | Yes | 7 | Low |
| Cetin, En. et al. (2022) | No | Yes | No | Yes | Yes | Yes | Yes | Yes | Yes | Yes | 8 | Low |
| Sen, En. et al. (2018) | No | Yes | No | Yes | Yes | Yes | Yes | Yes | Yes | Yes | 8 | Low |
| Cai, Y. et al. (2022) | No | Yes | No | Yes | Yes | Yes | Yes | Yes | Yes | Yes | 8 | Low |
| Cankaya, C. et al. (2018) | No | Yes | No | Yes | Yes | Yes | Yes | Yes | Yes | Yes | 8 | Low |
| Ozdamar, Y. et al. (2010) | No | Yes | No | Yes | Yes | Yes | Yes | Yes | Yes | Yes | 8 | Low |
| Agra, C. et al. (2014) | No | Yes | No | Yes | Yes | Yes | Yes | Yes | Yes | Yes | 8 | Low |
| Sen, En. et al. (2015) | No | Yes | No | Yes | Yes | Yes | Yes | Yes | Yes | Yes | 8 | Low |
| Heinz, C. et al. (2012) | No | Yes | No | Yes | Yes | Yes | Yes | Yes | Yes | Yes | 8 | Low |
| Szepessy, Z. et al. (2016) | No | Yes | No | Yes | Yes | Yes | Yes | Yes | Yes | Yes | 8 | Low |
| Dikmetas, O. et al. (2022) | No | Yes | No | Yes | Yes | Yes | Yes | Yes | Yes | Yes | 8 | Low |
| Ozer, M. et al. (2019) | No | Yes | No | Yes | Yes | Yes | Yes | Yes | Yes | Yes | 8 | Low |
| Cankaya, C. et al. (2015) | No | Yes | No | Yes | Yes | Yes | Yes | Yes | Yes | Yes | 8 | Low |
| Mocan, M. et al. (2011) | No | Yes | No | Yes | Yes | Yes | Yes | Yes | Yes | Yes | 8 | Low |
| Setälä, K. et al. (1979) | No | Yes | No | Yes | Yes | Yes | Yes | Yes | Yes | Yes | 8 | Low |
| Turan-Vural, En. et al. (2012) | No | Yes | No | Yes | Yes | No | Yes | Yes | Yes | Yes | 7 | Low |
| Choi et al. (2016) | Yes | Yes | No | Yes | Yes | yes | Yes | Yes | Yes | Yes | 9 | Low |

1. Was the study's target population a close representation of the national population in relation to relevant variables, e.g. age, sex, occupation?
2. Was the sampling frame a true or close representation of the target population?
3. Was some form of random selection used to select the sample, OR, was a census undertaken?
4. . Was the likelihood of non-response bias minimal?
5. Were data collected directly from the subjects (as opposed to a proxy)?
6. Was an acceptable case definition used in the study?
7. Was the study instrument that measured the parameter of interest (e.g. prevalence of low back pain) shown to have reliability and validity (if necessary)?
8. Was the same mode of data collection used for all subjects?
9. Was the length of the shortest prevalence period for the parameter of interest appropriate?
10. Were the numerator(s) and denominator(s) for the parameter of interest appropriate

**S3 Table 3. Risk of bias or determination of the quality of cases and controls.**

| Author, Year | 1 | 2 | 3 | 4 | 5 | Recomendation |
| --- | --- | --- | --- | --- | --- | --- |
| Yilmaz 2021 | Yes | Yes | Yes | Yes | Yes | Include |

1. Can we be confident in the assessment of exposure?
2. Can we be confident that cases had developed the outcome of interest and controls had not?
3. Were the cases (those who were exposed and developed the outcome of interest) properly selected?
4. Were the controls (those who were exposed and did not develop the outcome of interest) properly selected
5. Were cases and controls matched according to important prognostic variables or was statistical adjustment carried out for those variables?

**S4 Table 4. Risk of bias or quality ascertainment of cohorts.**

| Author, Year | 1 | 2 | 3 | 4 | 5 | 6 | 7 | 8 | Recomendation |
| --- | --- | --- | --- | --- | --- | --- | --- | --- | --- |
| Ghiță, 2019 | Yes | No | No | Yes | No | Yes | Yes | Yes | Include |

1. Was selection of exposed and non-exposed cohorts drawn from the same population?

2. Can we be confident in the assessment of exposure?

3. Can we be confident that the outcome of interest was not present at start of study?

4. Did the study match exposed and unexposed for all variables that are associated with the outcome of interest or did the statistical analysis adjust for these prognostic variables?

5. Can we be confident in the assessment of the presence or absence of prognostic factors?

6. Can we be confident in the assessment of outcome?

7. Was the follow up of cohorts adequate?

8. Were co-interventions similar between groups?
