# Supplementary material for: Quantitative changes in the corneal endothelium and central corneal thickness during anterior chamber inflammation: A systematic review and meta-analysis
Source: PLoS One. 2024 Jan 5;19(1):e0296784. doi: 10.1371/journal.pone.0296784 (PMC10769021; doi:10.1371/journal.pone.0296784)

**Supporting Information 4: Sensitivity analysis of endothelial cell parameters and central corneal thickness.**

**S4 Fig 1 Endothelial cell density**

**S4 Fig 1.1 Analysis of articles that could be included in the meta-analysis of endothelial cell density.**


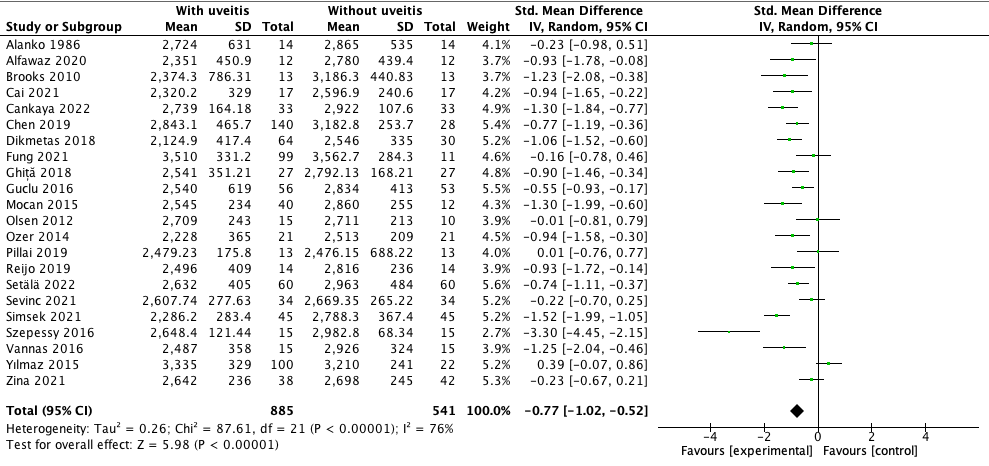


**S4 Fig 1.2 Sensitivity analysis of endothelial cell density filter for item quality.**


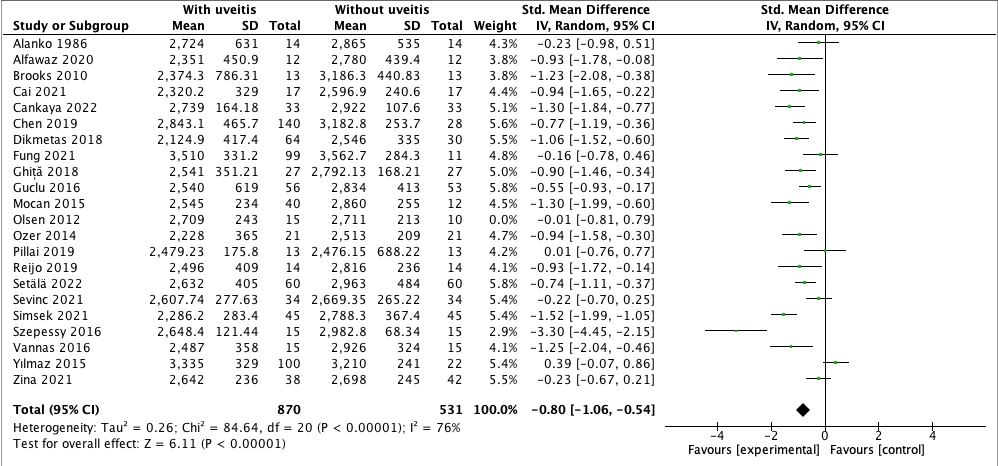


**S4 Fig 1.3 Sensitivity analysis of endothelial cell density filter outliers**


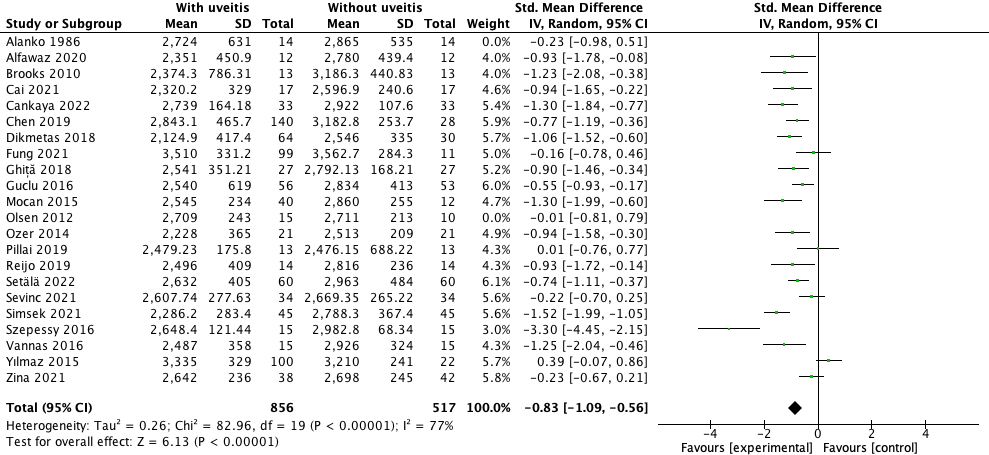


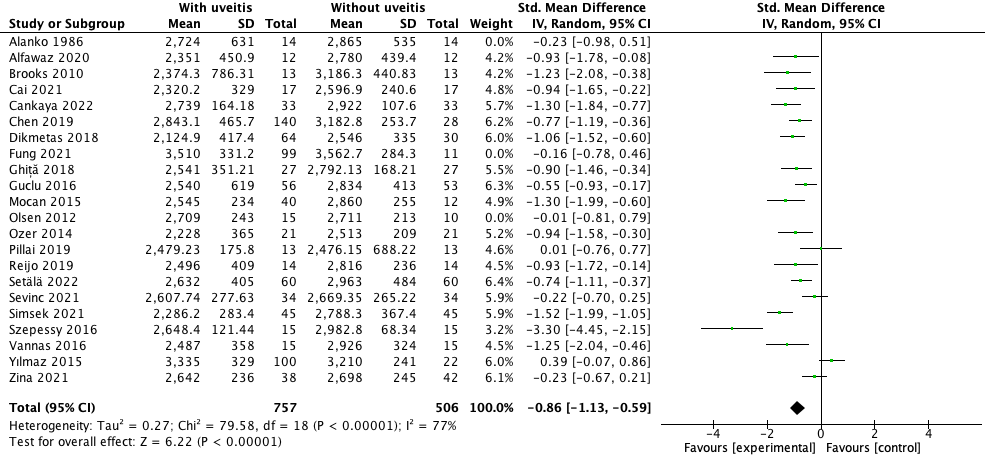


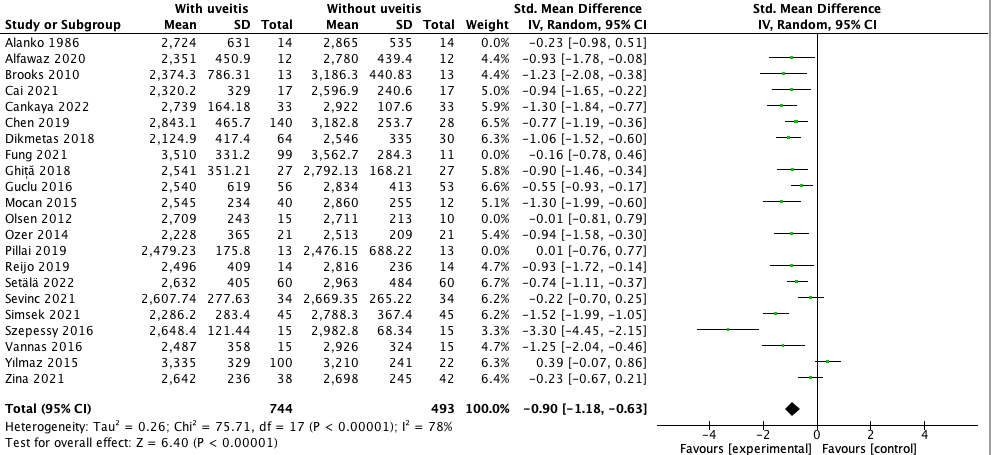


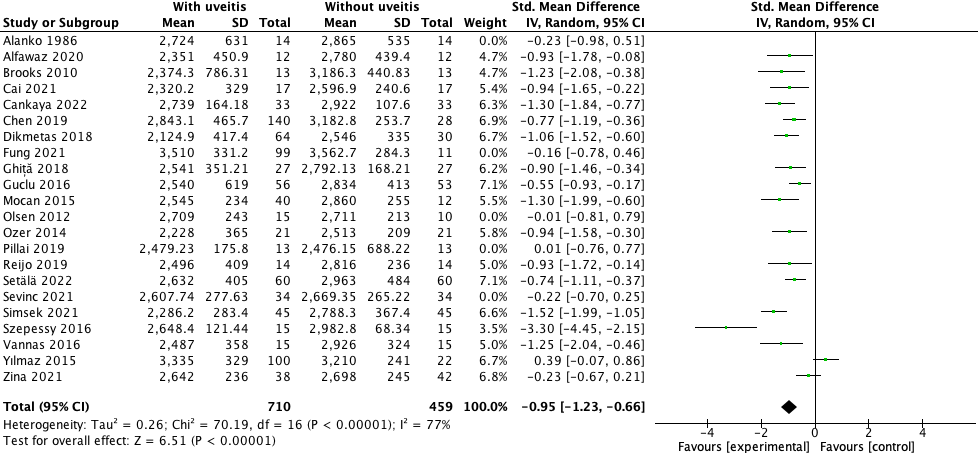

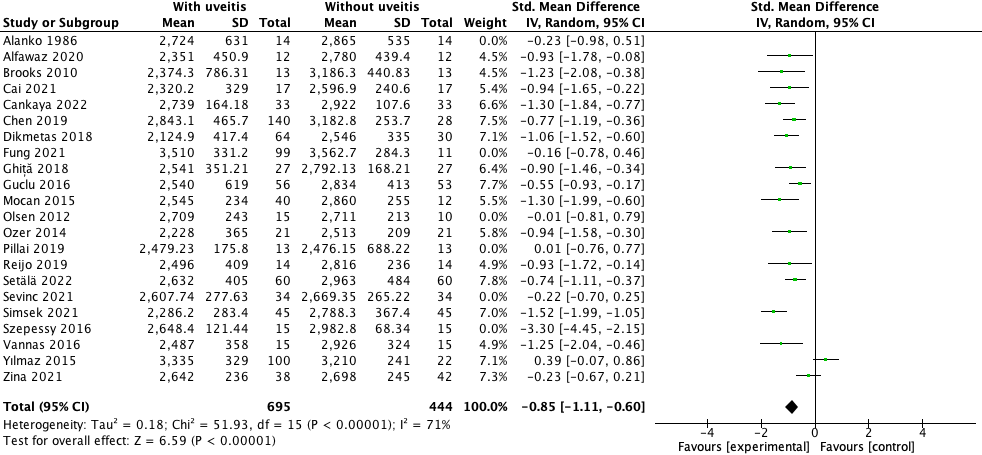


**S4 Fig 1.5 Analysis of articles likely to be included in the meta-analysis of endothelial cell density by subgroups of active vs. inactive uveitis**


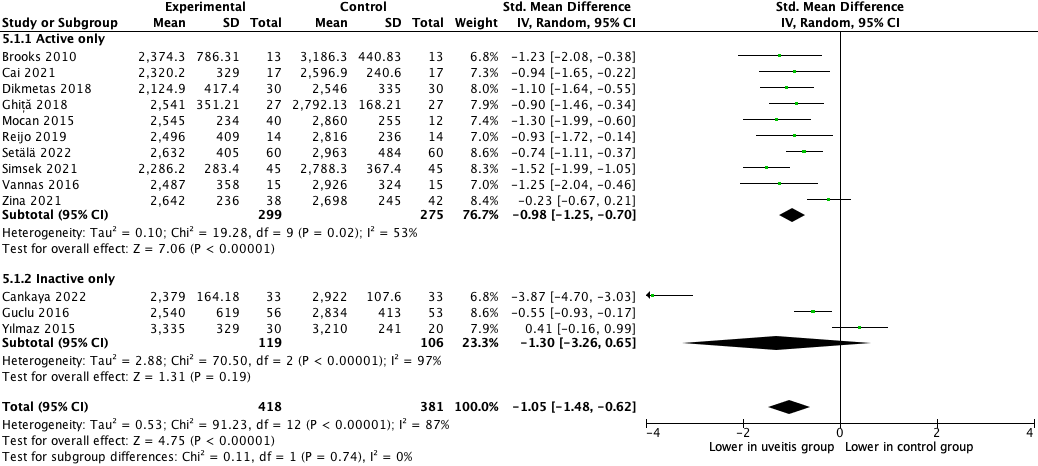


**S4 Fig 1.5 Sensitivity analysis of in the meta-analysis of endothelial cell density by subgroups of active vs. inactive uveitis filter outliers.
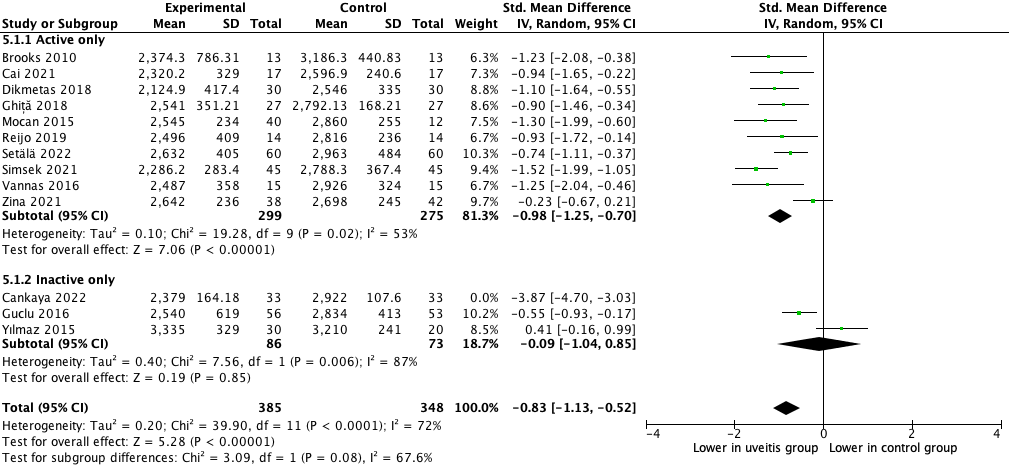
**

**S4 Fig 2 Coefficient of variation**

**S4 Fig 2.1 Analysis of articles that could be included in the coefficient of variation meta-analysis.**


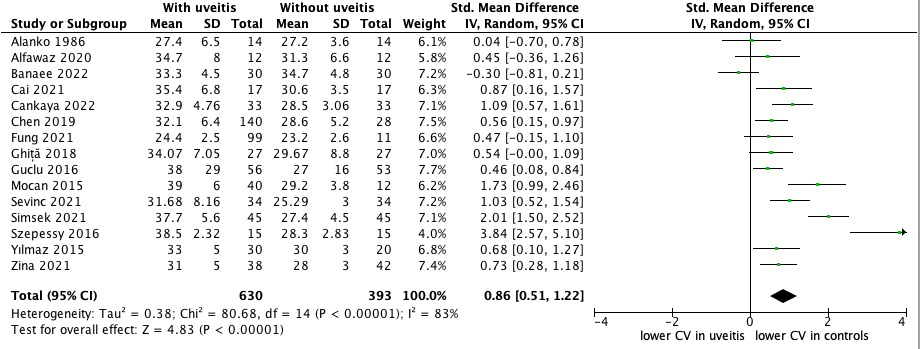


**S4 Fig 2.2 Sensitivity analysis of the coefficient of variation filter outliers**


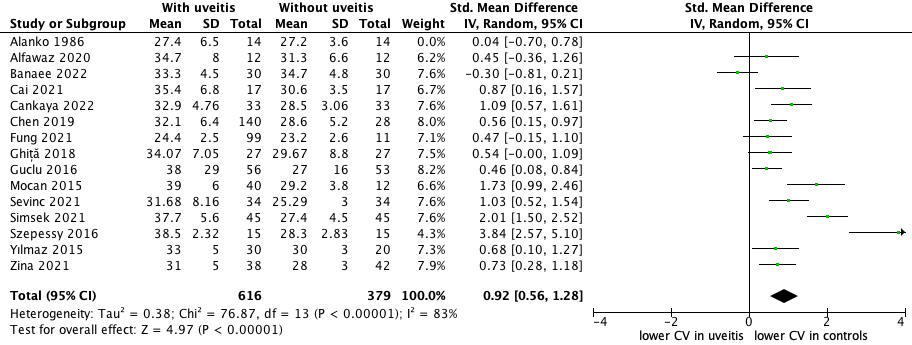


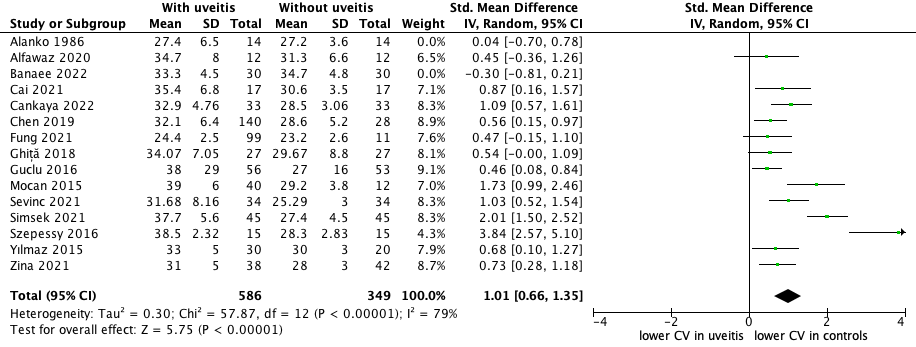


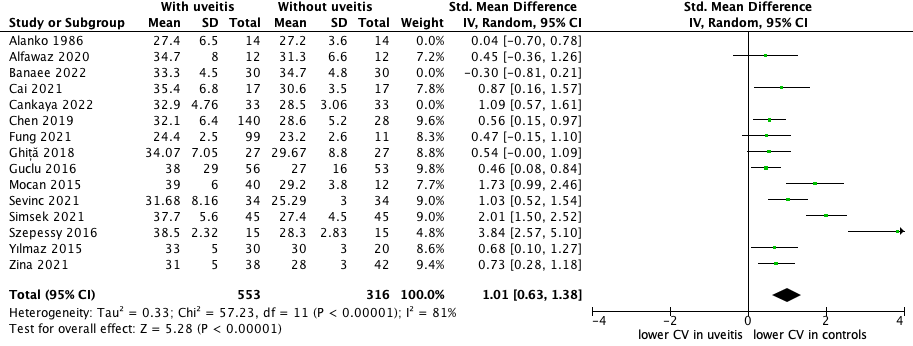


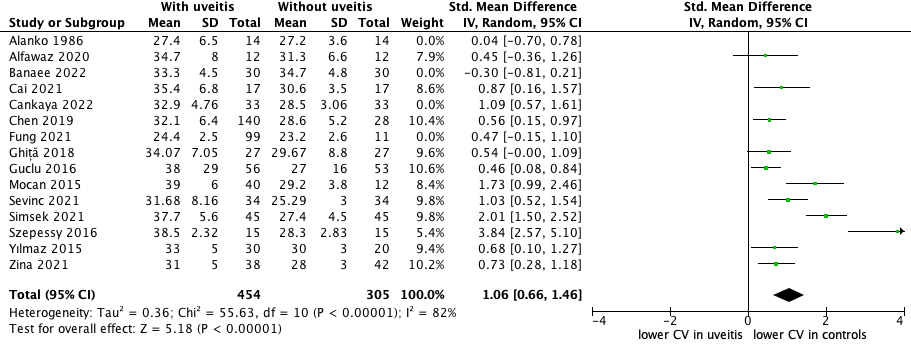


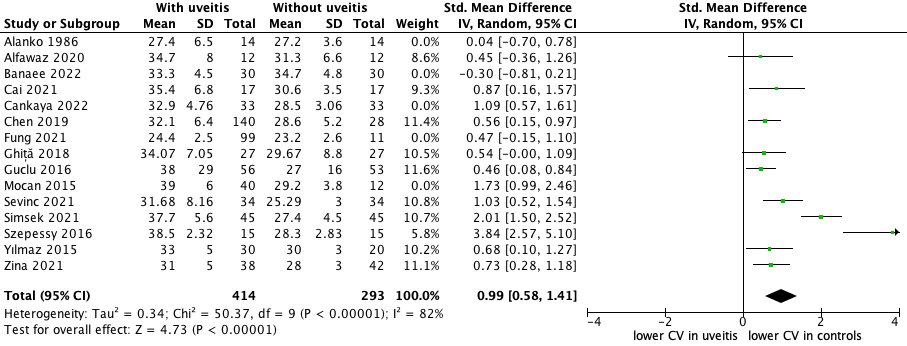


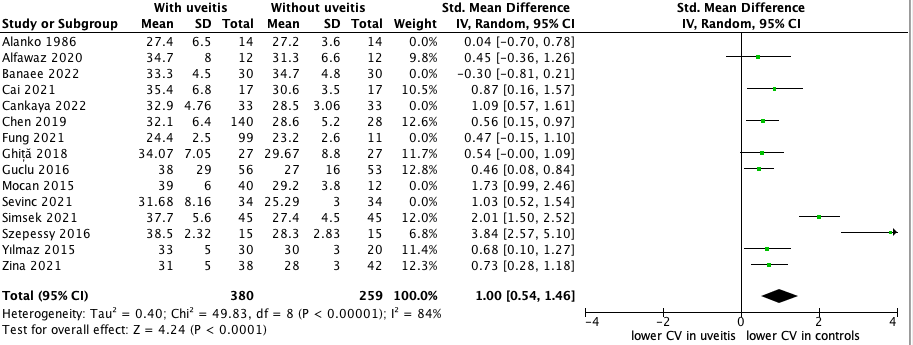


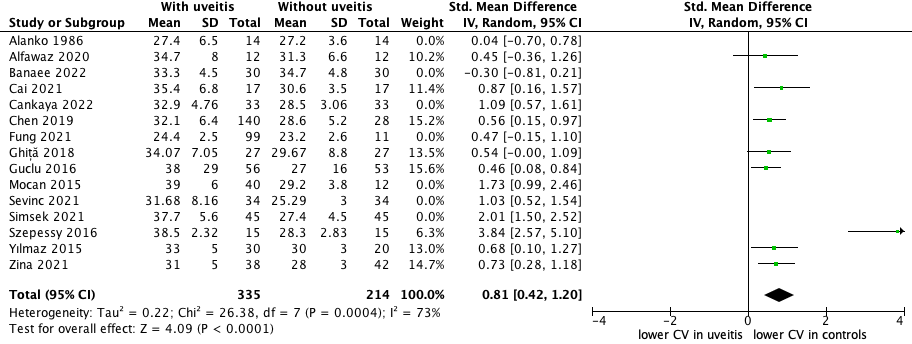


**S4 Fig 2.3 Analysis of articles likely to be included in the meta-analysis of the coefficient of variation by subgroups of uveitis course**


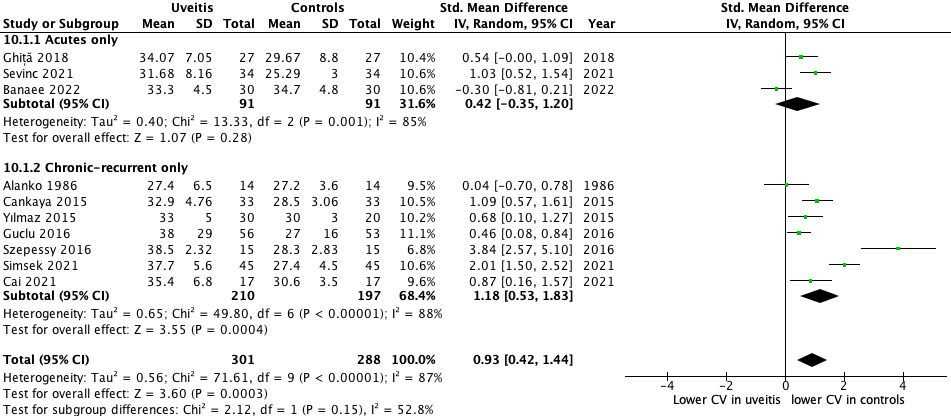


**S4 Fig 2.4 Sensitivity analysis of in meta-analysis of coefficient of variation by subgroups of uveitis course.**

**
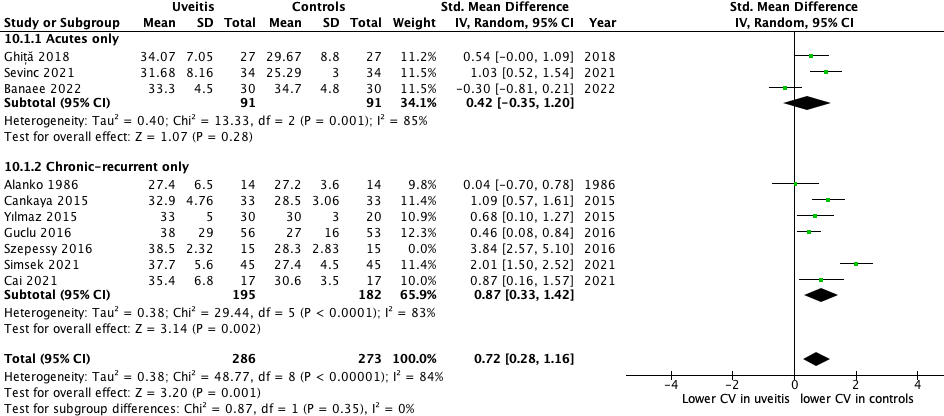
**

**S4 Fig 3 Hexagonality**

**S4 Fig 3.1 Analysis of articles that could be included in the hexagonal meta-analysis.**

**
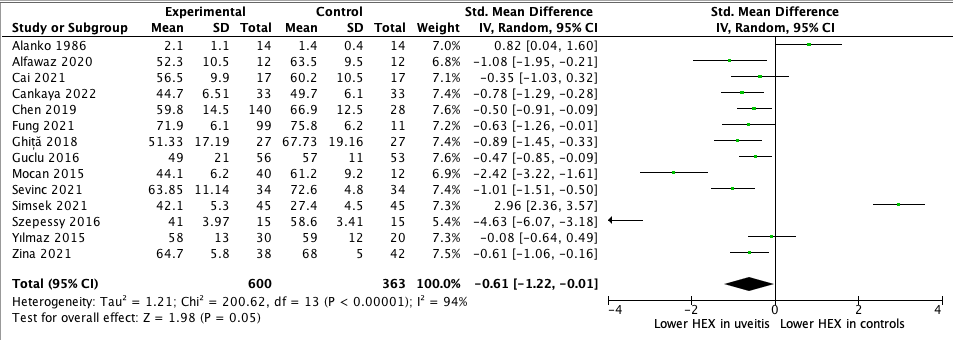
**

**S4 Fig 3.2 Sensitivity analysis of hexagonality filter outliers**
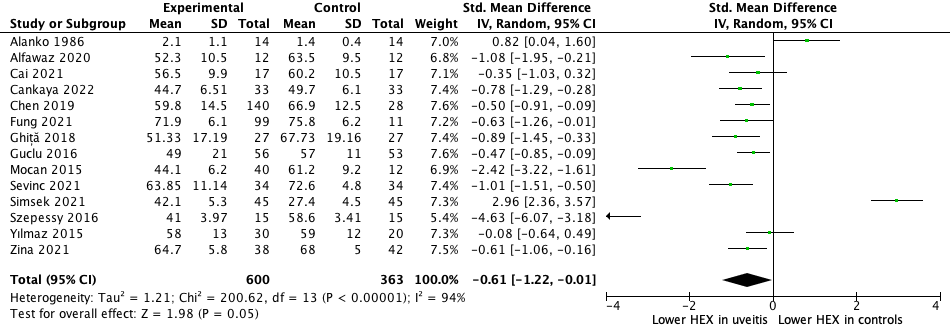


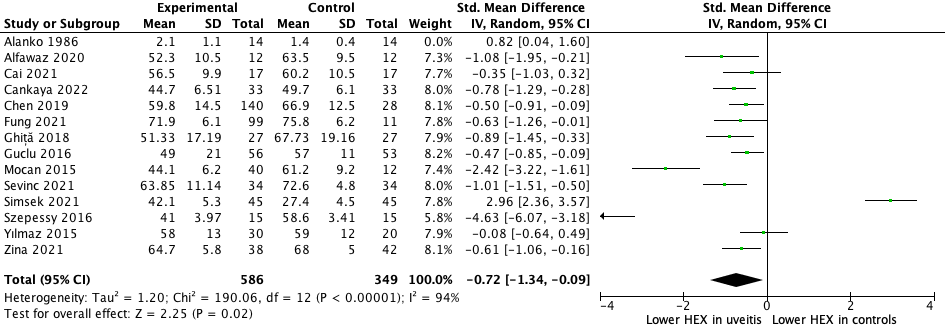


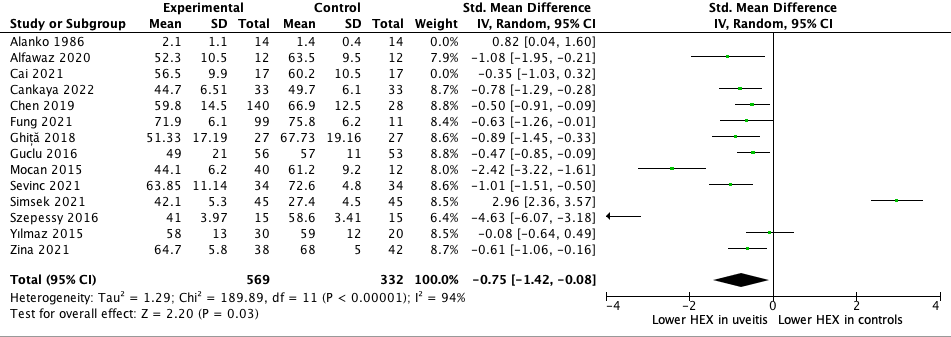

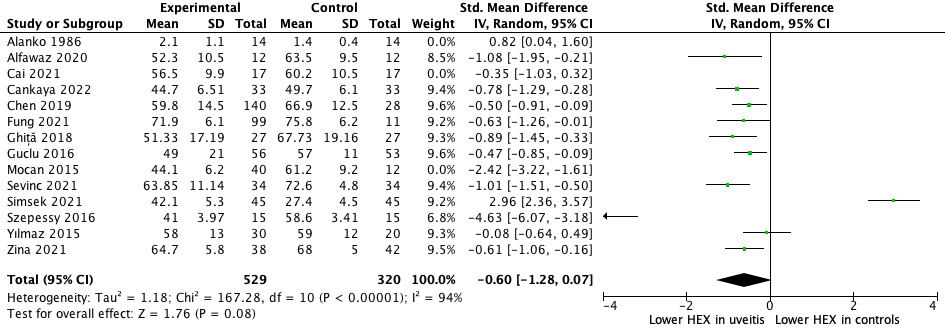

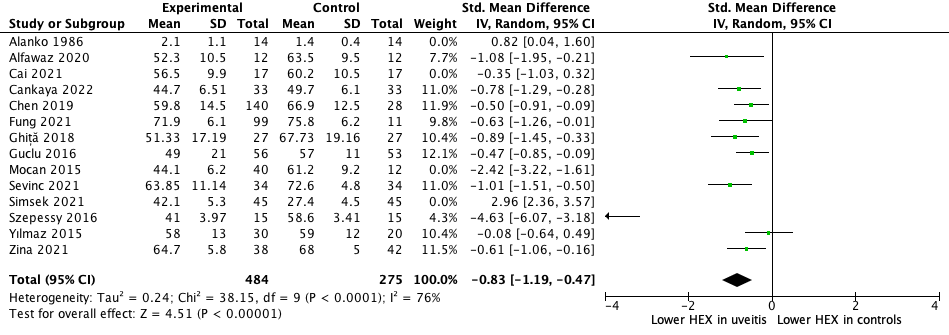

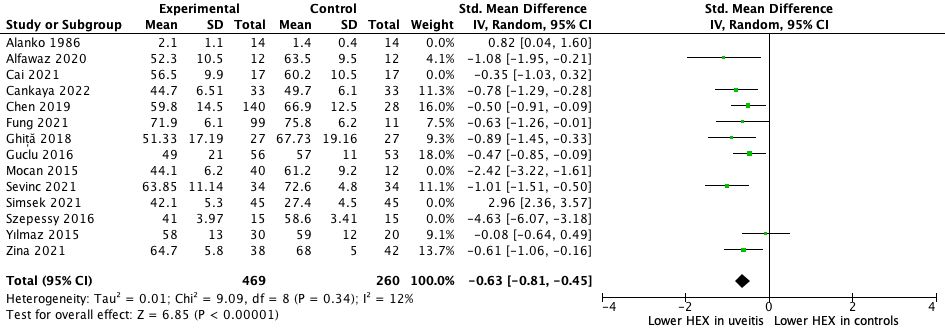


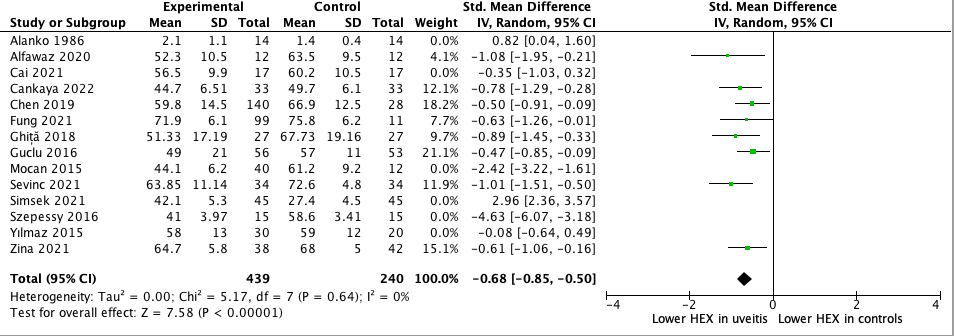


**S4 Fig 4 Central corneal thickness**

**S4 Fig 4.1 Analysis of articles that could be included in the meta-analysis of corneal thickness.**


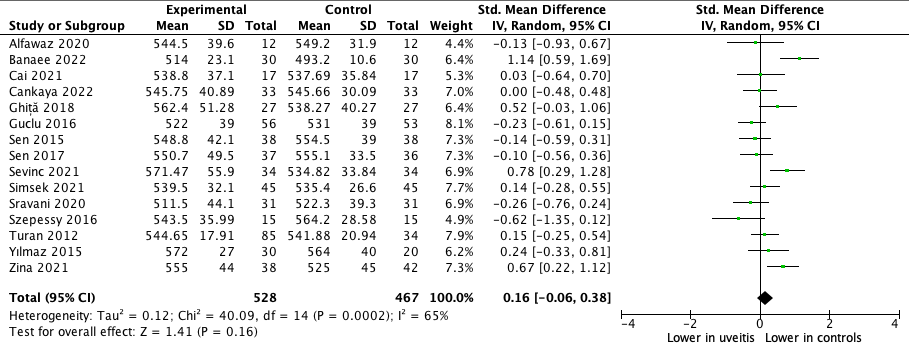


**S4 Fig 4.2 Sensitivity analysis of corneal thickness filter outliers**


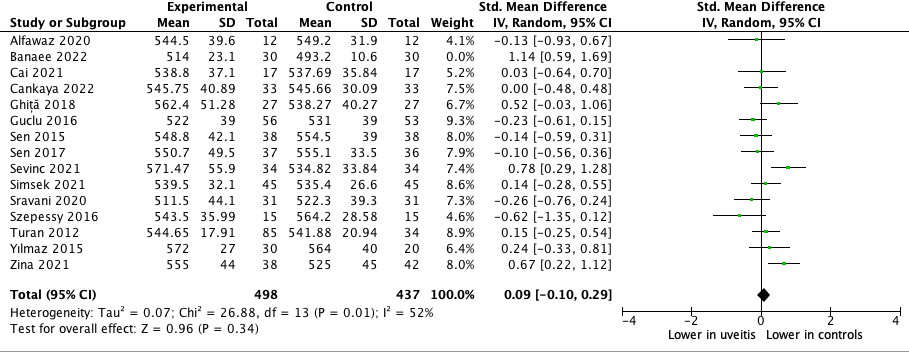


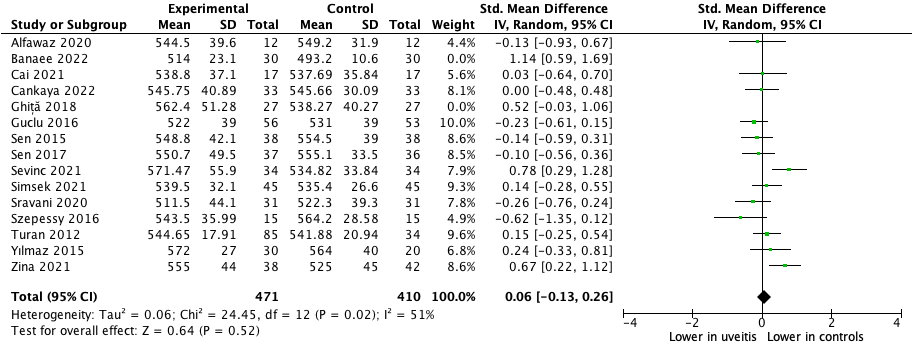


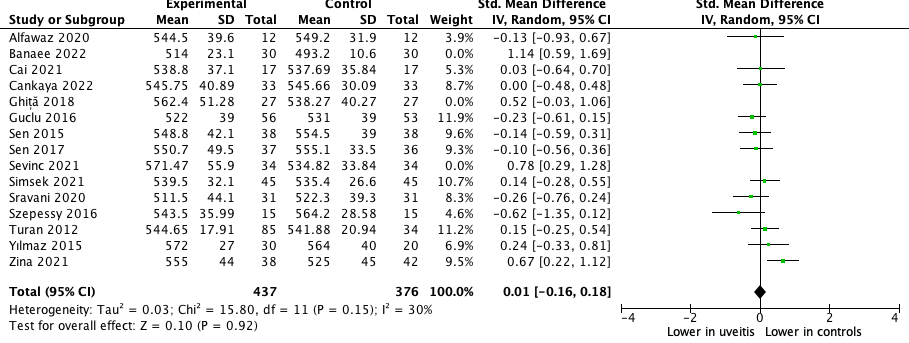

Supplement: S4 File — (DOCX) [file pone.0296784.s004.docx]
